# Advancing Applications of Black Phosphorus and BP‐Analog Materials in Photo/Electrocatalysis through Structure Engineering and Surface Modulation

**DOI:** 10.1002/advs.202001431

**Published:** 2020-08-06

**Authors:** Teng Yin, Liyuan Long, Xian Tang, Meng Qiu, Weiyuan Liang, Rui Cao, Qizhen Zhang, Dunhui Wang, Han Zhang

**Affiliations:** ^1^ School of Electronics and Information Hangzhou Dianzi University Hangzhou 310018 China; ^2^ Institute of Microscale Optoelectronics Collaborative Innovation Centre for Optoelectronic Science & Technology Key Laboratory of Optoelectronic Devices and Systems of Ministry of Education and Guangdong Province College of Physics and Optoelectronic Engineering Shenzhen Key Laboratory of Micro‐Nano Photonic Information Technology Guangdong Laboratory of Artificial Intelligence and Digital Economy (SZ) Shenzhen University Shenzhen 518060 China; ^3^ School of Physics and Optoelectronic Engineering Foshan University Foshan 528000 China; ^4^ Key Laboratory of Marine Chemistry Theory and Technology (Ocean University of China) Ministry of Education Qingdao 266100 P. R. China; ^5^ Advanced Institute of Information Technology Peking University Hangzhou 311215 China

**Keywords:** black phosphorus, black‐phosphorus‐analog materials, electrocatalysis, photocatalysis

## Abstract

Black phosphorus (BP), an emerging 2D material semiconductor material, exhibits unique properties and promising application prospects for photo/electrocatalysis. However, the applications of BP in photo/electrocatalysis are hampered by the instability as well as low catalysis efficiency. Recently, tremendous efforts have been dedicated toward modulating its intrinsic structure, electronic property, and charge separation for enhanced photo/electrocatalytic performance through structure engineering. Simultaneously, the search for new substitute materials that are BP‐analogous is ongoing. Herein, the latest theoretical and experimental progress made in the structural/surface engineering strategies and advanced applications of BP and BP‐analog materials in relation to photo/electrocatalysis are extensively explored, and a presentation of the future opportunities and challenges of the materials is included at the end.

## Graphical abstract

Black phosphorus (BP) shows great potential in photo/electrocatalytic applications. The advantages, limitations, modulation mechanism, and strategies of BP and BP‐based catalysts in the photo/electrocatalysis field are profoundly elucidated. Further, newly emerged BP‐analog materials are summarized and future directions for BP and BP‐analog materials in this field are provided.

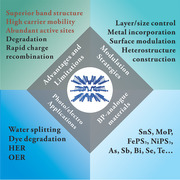

## 1 Introduction

Layered 2D materials with ultralarge specific area and ultrashort carrier diffusion distance have already attracted tremendous attentions in the catalysis field including photocatalysis,^[^ ^1^ ^]^ electrocatalysis,^[^ ^2^ ^]^ and photoelectrocatalysis.^[^ ^3^ ^]^ Toward the most type of 2D materials, changing from bulk to few‐layer can induce surprising variations in their intrinsic electronic and optoelectronic properties such as the widening bandgap owing to the decreased inter layer coupling and even the conversion from indirect bandgap to direct bandgap for certain materials.^[^ ^3^, ^4^ ^]^ One of the most famous metal‐free 2D materials is graphene with large surface area (theoretically 2630 m^2^ g^−1^) to induce more surface active sites and high carrier mobility to facilitate electron transfer, both of which benefit for the photo/electrocatalysis.^[^ ^5^ ^]^ However, graphene, as a semimetal 2D material, its zero‐bandgap severely limits its application in photocatalysis.^[^ ^3a^ ^]^ Thus, another 2D material, graphitic carbon nitride (g‐C_3_N_4_) with about 2.7 eV inborn bandgap inspires much more interest owing to its suitable valence band position and conduction band position for photocatalytic water splitting which transfers solar energy to chemical energy.^[^ ^6^ ^]^ Although it possesses excellent stability in both acidic and alkaline solutions, lacking surface active sites and low carrier separation efficiency both limit its applications as catalyst. Excavating more promising 2D materials which can be widely applied in photocatalysis and electrocatalysis is quite essential for dealing with energy shortage and waste water problems.

Since 2014, black phosphorus (BP) as the most stable allotrope of phosphorus with 2D sheet‐like structure similar to graphene has inspired much research interest. As a metal‐free semiconductor, BP exhibits layer‐dependent direct bandgap (0.3–2 eV) and in‐plane anisotropy different from graphene, which are in favor of excellent applications in solar energy storage,^[^ ^7^ ^]^ pollutant degradation,^[^ ^8^ ^]^ and optoelectronic devices.^[^ ^9^ ^]^ Besides, few‐layer BP prepared by mechanical and liquid exfoliation owns high carrier mobility and specific surface area respectively inducing facilitated carrier migration and increased surface active sites, thereby boosting photo/electrocatalysis. Toward photocatalysis, a wide range of sunlight absorption even extending to near‐infrared (NIR) wavelength which occupies 50% of solar light endows BP evident advantages over classic g‐C_3_N_4_.^[^ ^10^ ^]^ Although theoretical prediction about the potential of BP as a suitable photo/electrocatalyst has been reported for a long time, only a few reports focused on the experimental characterization and demonstration before 2016.^[^ ^2^, ^11^ ^]^ The nanohybrids assembling BP with TiO_2_ applying in the degradation of Rhodamine B (RhB) was the initial report about its photocatalytic performance in experiment.^[^ ^11a^ ^]^ The research bottlenecks are the difficulties of achieving high‐quality BP in large scale and the instability of BP in water and oxygen environment especially under light illumination. These problems are quite challenging for catalytic applications including photo/electrocatalytic water‐splitting for hydrogen and oxygen evolution, organic dye, or heavy metal degradation in polluted water and nitrogen fixation, which are inevitably involved by water.

After Yang's groups achieved visible‐light photocatalytic hydrogen evolution from pure BP nanosheets for the first time in 2017 (the hydrogen evolution rate of 512 µmol h^−1^ g^−1^ higher than g‐C_3_N_4_),^[^ ^12^ ^]^ more researchers focus on overcoming the initial limitations and enhancing the catalytic performance of BP through structure engineering. Bulk BP has been exfoliated into few‐layer BP nanoflakes and quantum dots from “top‐down” approaches including liquid exfoliation, ball‐milling method, and ion‐intercalation in high yield.^[^ ^13^ ^]^ Through controlling the exfoliation/ion‐intercalation solvent or ball‐milling additive, not just the exfoliated layer number and lateral size can be adjusted, but also the surface/edge structures can be exactly modified to promote the stability and even provide suitable active sites for hydrogen/oxygen evolution.^[^ ^12^, ^14^ ^]^ However, “bottom‐up” preparation method widely used in layered 2D materials has been rarely reported in BP nanostructure, owing to the rigorous high pressure and temperature for the phase change from red phosphorus to BP. Recently, BP nanosheets have been successfully prepared from a solvothermal reaction of white phosphorus in ethylenediamine.^[^ ^15^ ^]^ Such facile “bottom‐up” method is more conducive to achieve metal atom doping as well as other structure engineering strategies including molecule modification and heterointerface architecting.

Deeply understanding the intrinsic band structure as well as electronic properties of BP and rationally modulate BP through structure engineering really make sense for enhancing photo/electrocatalytic performance and even exploiting wider applications such as hydrogen evolution from pure water without sacrificial reagents.^[^ ^16^ ^]^ Herein, we first analyze the advantages and disadvantages of the intrinsic characteristics of BP in photo/electrocatalysis field. Second, based on its limitations, the recent strategies to modify the intrinsic structures, modulate the electronic properties, construct efficient charge separation, and achieve superior catalytic activity have been analyzed and comprehended here in detail. The kinetic mechanisms of sharply enhanced photo/electrocatalytic capability of BP have been classified to unravel for further application. Third, in addition to hydrogen evolution and RhB degradation, BP nanostructures have been widely applied in many other catalysis‐related fields and we thoroughly concluded here. Fourth, we introduce the properties and expect the catalytic applications for BP‐analog materials with similar layered and puckered 2D structures. Finally, we give an outlook for the future direction.

## 2 Advantageous Properties and Limitations in Photo/Electrocatalysis

### 2.1 Advantageous Properties

#### 2.1.1 Superior Band Structure

**Figure 1:**
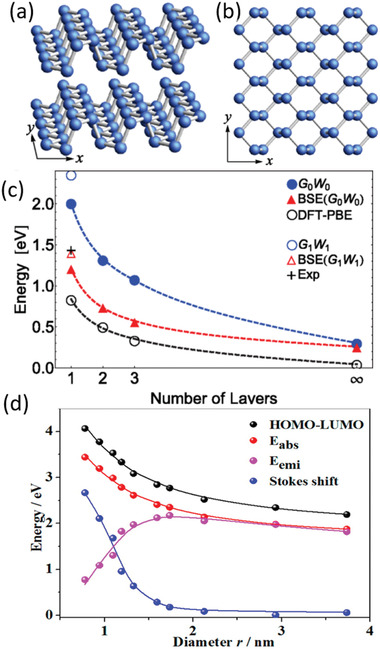
a,b) Ball‐stick models of the side view of few‐layer BP (a) and the top view of monolayer BP (b). The *x* and *y* axes are, respectively, along armchair and zigzag direction. c) The bandgap evolution of BP along with the layer number according to different calculation methods and optical absorption. a–c) Reproduced with permission.^[^ ^10a^ ^]^ Copyright 2014, American Physical Society. d) The calculated HOMO–LUMO energy gap, absorption gap, emission gap, and Stokes shift of BP quantum dots as a function of its diameter. Reproduced with permission.^[^ ^22^ ^]^ Copyright 2016, American Chemical Society.

Each layer of BP exhibits puckered structure in its armchair direction and bilayer configuration along the zigzag direction (**Figure** **1**a,b).^[^ ^17^ ^]^ The interaction between these puckered honeycomb layers is the van der Waals force. The special anisotropy 2D structure induces many unique properties including layer‐dependent and size‐dependent direct bandgap and anisotropic optical absorption from visible light to mid‐infrared.^[^ ^10b^ ^]^

Toward photocatalytic application especially solar energy conversion, infrared sunlight which is the majority section of sunlight should be effectively absorbed and utilized to induce electron transition. Different from most wide‐band semiconductors including TiO_2_,^[^ ^18^ ^]^ In_2_O_3_,^[^ ^19^ ^]^ g‐C_3_N_4_ using ultraviolet‐light, and partial visible‐light, multilayer‐BP has intensely absorption in infrared wavelength.^[^ ^10b^ ^]^ Unlike the typical transition metal sulfide MoS_2_ exhibiting a band structure reversion from indirect to direct one with reducing layer‐number, the always direct bandgap regardless of layer‐number endows BP a relatively high photoexcitation efficiency especially under near‐infrared (NIR) illumination whether being peeled into monolayer or not. Nevertheless, the magnitude of energy gap of BP evidently changes from bulk to monolayer as shown in Figure 1c.^[^ ^10a^ ^]^ This kind of layer‐dependent feature should be attributed to the altered charge distribution on each P atom arising from the interlayer van der Waals interactions.^[^ ^20^ ^]^ According to the ab initio calculations, totally different from graphene whose relative bands exclusively determined by *p_z_* orbital, toward BP, mixture of states of different symmetry make contributions to the formation of valence band (VB) and conduction band (CB) at the Γ point, leading to the less trivial band structure.^[^ ^21^ ^]^ It has been demonstrated that the van der Waals interaction induced by additional BP layer can result in band splitting over the entire Brillouin zones, leading to the increasing bandgap along with the decreasing thickness. Such adjustable CB and VB not only facilitate BP to construct heterostructure with different catalyst, but also to satisfy the essential reduction potential (H^+^/H_2_: 0.0 eV vs NHE at pH = 0) for hydrogen evolution in photocatalysis and photoelectrocatalysis.^[^ ^20^ ^]^ Besides the layer number, the optoelectronic properties of BP are also intimately related to the size.^[^ ^14b^ ^]^ According to time‐dependent density functional theory (TDDFT), the bandgap BP quantum dots satisfy an inversely proportional law of *A* + *B/r* ^2^ + *C/r* owing to the quantum confinement effect.^[^ ^22^ ^]^ Thus, the electronic bandgap (highest occupied molecular orbital (HOMO)–lowest unoccupied molecular orbital (LUMO) gap) of BP reduces sharply along with the increasing size as shown in Figure 1d. As the bandgap of BP is quite sensitive to thickness and size, most of the prepared BP nanomaterials always display a wide range of optical absorption in the whole wavelength of sunlight, which further enhances the solar utilization efficiency.

Another typical feature of BP is the high in‐plane anisotropy, which results from its antisymmetric and mirror reflection symmetric crystal structure.^[^ ^23^ ^]^ This intrigues the highly asymmetric band structure and direction‐dependent optical absorption for few‐layer BP.^[^ ^23b^ ^]^ In other words, the absorption coefficient of such 2D BP strongly depends on the polarization state of incident light. According to the theoretically calculated optical absorption by Qiao et al., optical band edges of BP always changes with increasing layer number no matter for armchair direction and zigzag direction, while the fall rate and the band‐edge energy of these two orientations differ largely from each other.^[^ ^24^ ^]^ Owing to the much lower band edge for armchair orientation, the polarized visible light will tend to adsorb by armchair orientation instead of zigzag orientation.^[^ ^25^ ^]^ The unique anisotropic band structure can result in the polarized‐light‐dependent photocatalytic property. Furthermore, the recombination efficiency of photogenerated carriers is negatively related to the photon diffusion coefficient. Considering the 10‐times larger coefficient absorption and 16‐times smaller photodiffusion of the zigzag direction compared to the armchair direction,^[^ ^26^ ^]^ regulating the polarization orientation of incident light such as introducing microgrid mode resonance structure may not merely enhance the photo absorption, but also inhibit the fast charge recombination as well as prolong the lifetime of energetic electrons for enhanced quantum efficiency of BP. These results deeply unravel the band structure superiorities of BP for applying in photocatalysis.

#### 2.1.2 High Carrier Mobility and Good Conductivity

One of the most attractive characters for BP applying as catalyst is the extremely high carrier mobility.^[^ ^27^ ^]^ Predicted by Qiao's calculation adopting a phonon‐limited scattering model, the hole mobility and electron mobility of BP can reach up to 10 000–26 000 and 1100–1140 cm^2^ V^−1^ s^−1^, respectively, which even parallel with graphene and much higher than 2D TMDs.^[^ ^24^ ^]^ Owing to the directional anisotropy of BP, its carrier mobility exhibits moderately in‐plane anisotropy. The hole mobility for the *y* direction is enhanced to be much larger than *x* direction when the layer number decreases to single, while it is almost twice smaller than *x* direction in several‐layer BP. For electrons, the *x* direction is always more in favor of mobility than *y* direction. Besides, the holes are more mobile than electrons in both directions. Such evident difference between hole and electron mobility should arise from their deformation potential, which dependent on the overlap of wavefunctions respectively for VB and CB. The absence of interlayer and stacking‐induced intralayer overlap for VB wave‐functions in monolayer BP induces the extremely small deformation potential along *y* direction, resulting in such excellent hole mobility. These features are extremely beneficial for the separation of holes and electrons and promoting the charge migration to the surface active sites for triggering redox reaction.

Although the electronic transport properties of BP in experiment are not as ideal as theory, numerous reports have corroborated the excellent carrier mobility for few‐layer BP. As reported by Li et al., the hole mobility for pure 10 nm thick BP nanoflakes detected through fabricating field‐effect transistor can reach 1000 cm^2^ V^−1^ s^−1^.^[^ ^7e^ ^]^ A high electron mobility of up to 2140 cm^2^ V^−1^ s^−1^ was achieved by electron doping with Cu adatoms.^[^ ^28^ ^]^ Moreover, our team has acquired high quality nanoflakes through hydrogenation to remove lattice oxygen atoms to repair phosphorous vacancies induced by mechanical exfoliation and hydrogenation.^[^ ^29^ ^]^ Such few‐layer BP exhibits high ambipolar field‐effect mobility of 1374 cm^2^ V^−1^ s^−1^ for holes and 607 cm^2^ V^−1^ s^−1^ for electrons, which are totally consistent with the more mobile feature of holes in the theoretical prediction. Although the experimental results for electronic properties of BP exhibit somewhat disparity with theoretical expectation, the similar intrinsic features of its carrier mobility are still conducive to charge transfer and separation. Actually, the certain gap of such carrier mobility should be attributed to the interface resistance in detection, the inevitable lattice defects and uncontrollable layer number in preparation. Thus, quite number of researches focus on preparing more homogeneous and thinner few‐layer BP to apply in catalysis.

#### 2.1.3 Active Sites

Few‐layer BP is commonly regarded as a semiconductor full of abundant active sites which are essential for completing the adsorption, activation, and reaction procedures in catalysis field.^[^ ^27^, ^30^ ^]^ These active sites are not only attributed to the 2D structure with large surface area, but also intimately related to the uncoordinated lone pair electrons of P atom and unavoidable point defects including P dangling bonds, the diagonal oxygen bridge (P—O—P) and the most stable dangling oxygen.^[^ ^14^, ^27^, ^31^ ^]^ Unlike another metal‐free semiconductor graphitic carbon nitride which lacks intrinsic active sites and need to enhance catalytic activity through heteroatom doping and surface defect groups (e.g., —NH_2_ group) introduction, few‐layer BP possesses inborn catalytic activity and can be even adopted as cocatalyst with g‐C_3_N_4_, Bi_2_WO_6_, and other relatively inert catalysts.^[^ ^32^ ^]^

**Figure 2:**
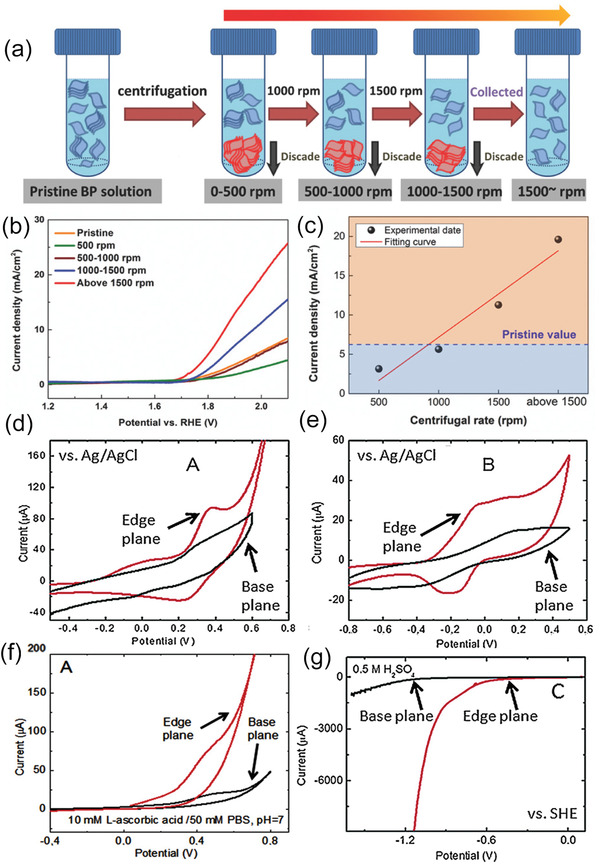
a) Schematic illustration of thickness‐selective process of BP nanosheets through centrifugation. b) Polarization curves and c) current density at 1.8 V of BP acquired from different centrifugation speeds. a–c) Reproduced with permission.^[^ ^30^ ^]^ Copyright 2017, Wiley‐VCH. d,e) Cyclic voltammograms with Fe complex (d) and Re complex (e) as the redox probe for basal‐plane and edge‐plane BP electrodes. f) The activity of basal‐ and edge‐plane BP toward oxidation of ascorbic acid. g) The HER reaction on basal‐ and edge‐plane BP electrodes. d–g) Reproduced with permission.^[^ ^34^ ^]^ Copyright 2016, Wiley‐VCH.

Recent advances manifested that reducing the thickness or size of BP would introduce additional exposed active sites to accommodate the adsorption and desorption processes.^[^ ^30^, ^33^ ^]^ Zhang and co‐workers reported that the decreasing layer number of BP nanosheets would boost more active sites owing to the ultrathin lamellar structure and thereby contribute to the electrocatalytic activities.^[^ ^30^ ^]^ They used the facile liquid exfoliation method following with centrifugation to acquire BP nanosheets with quantified thickness, shown in **Figure** **2**a. As the thickness distribution of BP decreases with improving centrifugation speed, the current densities exhibited in the polarization curves are almost negatively correlated with the layer number of BP (Figure 2b,c). Besides, the Tafel slope of the ultrathin BP is evidently lower than the thick BP. The obvious enhanced electrochemical oxygen evolution reaction (OER) performance indicated that reducing the thickness is supposed to be an effective way to generate additional active sites and improve interaction area for OER process. The exfoliation‐induced additional exposed surface lone‐pair electrons and edge under coordinated P atoms should largely contribute to the increased catalytic active sites. As BP is a semiconductor with layered 2D structure, its catalytic activity difference of basal plane and edge plane should be taken into consideration. Sofer and co‐workers combined DFT calculations with experiments to deeply investigate the anisotropy of the electrochemical and electronic properties of BP with edge‐ and basal‐plane‐oriented surfaces.^[^ ^34^ ^]^ According to the ab initio calculation results, the edge plane exhibits a much higher surface energy of 194 mJ m^−2^ compared to the negligible 9 mJ m^−2^ of basal plane. Such energetic surface is more in favor of the adsorption of various species and thereby exhibits a promoted catalytic activity. Besides, the orientation of lone‐pair electrons of P atom outward from the surface can further accentuate the activity of edge sites. Their experimental results further demonstrated the theoretical calculations. As shown in Figure 2d,e, the basal‐plane and edge‐plane BP electrodes were investigated as redox probe, respectively, for Fe and Ru complexes. For both complexes, the oxidation and reduction peaks detected on the edge‐plane electrode are much more evident than the basal‐plane electrode, indicating that the edge sites are more active than basal sites for BP. Such tendency of BP was also observed for the oxidation of biological compounds (Figure 2f). Figure 2g shows the hydrogen evolution reaction (HER) results. The reduction reaction for edge‐plane BP began in a potential of −0.55 V much higher than basal plane while −1.13 V for basal plane. Such observed more excellent catalytic activity for edge sites should be attributed to the metallic character of the edge planes different from the semiconducting property of basal planes as predicted in ab initio calculations. According to these researches, we can deduce that the diminished lateral dimensions of BP nanosheets would induce increasing edge active sites and further facilitate the catalytic activity. Therefore, the BP quantum dots (BPQDs) with numerous edge active sites has been found to display enhanced catalytic activity no matter in electrocatalytic and photocatalytic HER.^[^ ^17^, ^35^ ^]^

### 2.2 Limitations

#### 2.2.1 Degradation under Oxygen, Water, and Light

Although few‐layer BP owns several vital superiorities including wide optical absorption, tunable band structure, high carrier mobility, and decent catalytic activity, lacking of durability in ambient environment still restricts its catalytic performance in energy conversion and pollutants degradation.^[^ ^36^ ^]^ As the most stable allotrope of phosphorus, BP is still inevitable to be degraded by oxygen and water especially under illumination, leading to the deterioration of crystal structure and optoelectronic properties. The physical changes of nonuniform areal and volumetric expansion will induce increased surface roughness and thereby lower carrier mobility.^[^ ^37^ ^]^ Quantities of surface oxygen defects induced by degradation can cause deep donor or acceptor levels in the bandgap. These complex defect states may trap photogenerated electrons, change the carrier migration, and even lead to lose photocatalytic ability for the energy level lower than the redox potential.^[^ ^37^ ^]^ To overcome such challenge, researchers are supposed to unravel the degradation mechanism and the interaction processes with oxygen, water molecules, and photoenergy.

**Figure 3:**
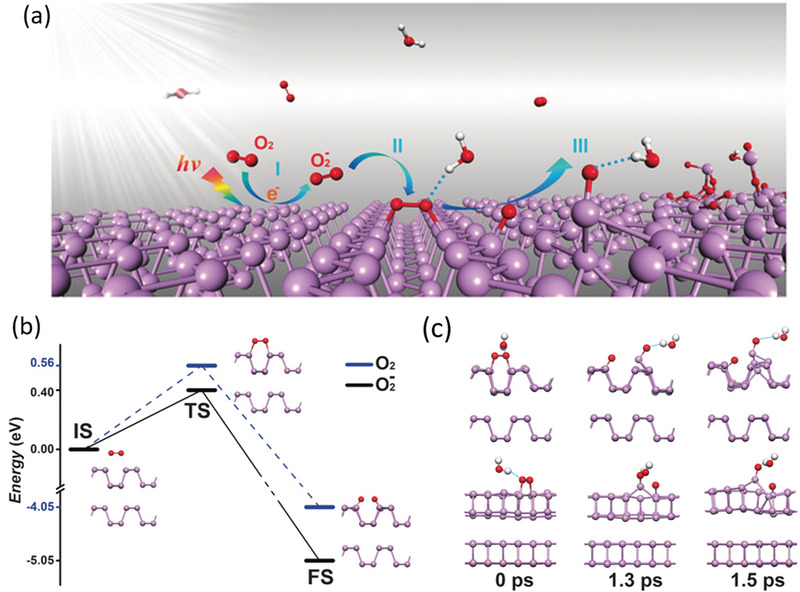
a) Schematic illustration of light‐induced ambient degradation of BP. I–III correspond to the three steps. b) The calculated reaction path for O_2_^−^ and O_2_ adsorbed on bilayer BP. c) The simulated dynamic process of H_2_O on oxygen adsorbed bilayer BP. The breaking of the P—P bond at the top layer induces the breakdown of BP after 1.5 ps. O red, H white, and P purple. a–c) Reproduced with permission.^[^ ^41^ ^]^ Copyright 2016, Wiley‐VCH.

The origin of the instability for BP is found to mainly emanate from the natural coordination structure of P atom and intrinsic defects introduced during crystal growth.^[^ ^38^ ^]^ The free lone‐pair electrons of each P atom due to sp^3^ hybridization result in the unstable bonding structure to be susceptible to chemisorbed oxygen atoms and then being oxidized. In general, the formation of full superficial oxide layer can be a passivation layer for preventing further oxidation of the inner structure such as Al_2_O_3_, while for BP, such protection will be destroyed by water molecules.^[^ ^39^ ^]^ Although the protection layer can be resumed with the cost of inner BP, the reserved crystallized BP will become less and less until all turn into H_3_PO_3_ and H_3_PO_4_. Actually, the surface oxidation of BP to form PO*_x_* could convert the pristine hydrophobic surface into hydrophilic and strongly facilitate the surface deterioration.^[^ ^40^ ^]^ Further researches have demonstrated that the ambient light induced surface photooxidation also plays a vital role in degradation process. Theoretical calculations have been used to deeply understand such ambient degradation mechanism.^[^ ^41^ ^]^ Three steps have been involved in this reaction as shown in **Figure** **3**a. First, photoinduced charge transfer reaction generates the superoxide anions on the BP surface. Second, the superoxide anions are dissociated into dangling oxygen atoms through a triplet‐singlet conversion (Figure 3b). Finally, H‐bonds of water molecules dissolve the dangling O and its bonded P from the crystallized BP (Figure 3c).^[^ ^41^ ^]^ Such process is dependent on the bandgap width and conduction band edge position which are respectively responsible for ambient light absorption and the energy gap with photoredox potential of generating superoxide anions. These calculation results are totally corresponding to the thickness‐dependent degradation characteristic of few‐layer BP.

Recently, some results have proved that intrinsic defects such as lattice oxygen defects and P vacancies introduced during crystal growth and mechanical exfoliation also play a significant role.^[^ ^42^ ^]^ Wu and co‐workers conducted scanning tunnel microscopy (STM) characterization with DFT calculations to prove the existence of lattice oxygen.^[^ ^29^ ^]^ The hydrogenation and phosphorization methods are conducted to remarkably enhance the stability of few‐layer BP. The chemical reactions are displayed in the following

(1) $$H_{2}+PO_{x}\to PH_{2x}+H_{2}O$$

(2) $$P_{y}+PH_{2x}\to P_{\mathrm{vacancy}}+PH_{3}$$

(3) $$P_{\mathrm{vacancy}}+P_{y}\to P_{\mathrm{pure}}$$

Here, the lattice oxygen defects (PO*_x_*) are removed after reacting with hydrogen and thereby P vacancies are formed. After phosphorization via vapor‐phase phosphorus clusters (P*_y_*), the P vacancies are repaired to acquire pure BP. The as‐treated few‐layer BP exhibits highly improved under ambient stability and carrier mobility (1374 cm^2^ V^−1^ s^−1^ for holes and 607 cm^2^ V^−1^ s^−1^ for electrons at 2 K). These results further manifest that the intrinsic defect is considered as one of the most essential challenges to achieve durability of BP.

Numerous efforts have been made to enhance the optoelectronic stability and catalytic recycle durability of BP,^[^ ^12^, ^36^, ^43^ ^]^ whereas boosting stability with physical encapsulations or surface passivation possibly become contradictory to catalytic activity, owing to hindering the charge transfer on the reaction interface and decreasing the active sites for species adsorption. Therefore, it is urgent to figure out a way not just protect BP from oxidation and dissolution, but also retain the superior catalytic activity of the surface. Recently, surface functionalization is adopted as an effective means for achieve the target.^[^ ^14^, ^44^ ^]^ Considering the exposed lone‐pair electrons of P atom being the major reason of degradation, occupying the lone pair with catalysis‐active metal atoms or functional groups to form the P—X bonds such as P—OH, P—NH_2_, P—Co, and P—Ni guarantees the surface active sites and catalytic durability of BP simultaneously.^[^ ^12^, ^14^, ^45^ ^]^ As a result, an appropriate metal atom or functional group to bond the lone pairs of P nearby the reaction interface should be more favorable in catalytic field.

#### 2.2.2 Rapid Charge Recombination

Toward photocatalytic reaction, the following steps are generally involved in semiconductor‐based system. First, light illumination induces the electron transition from VB to CB for generating the photoinduced electron–hole pairs. After that, these photoelectrons and photoholes respectively migrate to the surface active sites to trigger reduction and oxidation reactions.^[^ ^46^ ^]^ Therefore, once numerous photogenerated electrons recombine with holes before the diffusion to the active sites or undergoing redox reaction, the quantum efficiency and energy conversion efficiency will be sharply inhibited.

Actually, even though only a few reports exhibit intuitive evidences, there are quantities of reports indicating the fast recombination and ultrashort lifetime of photocarriers of BP.^[^ ^46^, ^47^ ^]^ Wang and co‐workers acquired a piece of BP from mechanically exfoliating bulk BP and demonstrate its photocarrier lifetime of about 100 ps.^[^ ^26^ ^]^ Recently, several researches adopted liquid‐exfoliation or ball‐milling method to prepare BP in large scale and took advantage of the transient absorption to characterize the lifetime of photoexcited carriers of these BP nanosheets and quantum dots.^[^ ^32^, ^46^, ^47^, ^48^ ^]^ Most of these reports indicate that the average lifetime of excited carriers is only several picoseconds. These differences in photocarrier lifetime possibly arise from the difference in crystalline as well as size and thickness owing to the totally diverse preparation methods. Nevertheless, there exists a strong nonradiative electron–hole recombination channel in BP is commonly accepted and such nonradiative recombination leads to a relatively short lifetime which goes against photocatalytic capability. Actually, as a layered 2D material, BP exhibit reduced quantum confinement effects and decreased bandgap along with the increased layer number. Simultaneously, the increased electron‐vibrational nonadiabatic couplings and accelerated decoherence are also induced by the additional interlayer interaction, leading to a shorter excited electron–hole lifetime.^[^ ^49^ ^]^ Thus, reducing layer number of BP may induce slow charge recombination. Recently, several reports suggested that the rational introduction of defects such as grain boundaries and vacancies may induce long‐lived free charge‐separated state to achieve superior photocatalytic devices.^[^ ^49^, ^50^ ^]^ According to these results, numerous strategies can be adopted to modulate the charge migration property and intrinsic photo/electronic characteristics of BP for regulating its photocatalytic activity.

## 3 Strategies of Structure Engineering and Surface Modulation to Enhance Photo/Electrocatalysis Properties

Recently, to take advantage of the intrinsic merits of BP and simultaneously overcome its limitations, numerous efforts have been dedicated into modulating its intrinsic structure, electronic property, and charge separation for enhanced photo/electrocatalytic performance through structure engineering. These engineering strategies can be classified into the following four types: thickness/size regulation, metal incorporation, surface covalent modification, and heterostructure construction. Although some of these methods possibly have been adopted into modifying other catalysts,^[^ ^51^ ^]^ the unique intrinsic property of BP will induce large discrepancy in the catalytic activation mechanism. In this section, we will introduce the mechanism of photo/electrocatalysis in convenient of understanding the modification strategies. Then, we will not merely conclude the methods to acquire enhanced BP‐based catalysts, but more focus on analyzing the inherent kinetic mechanisms such as how to achieve charge separation in the real space and how to activate the initial catalytic active sites, thereby paving a way for the further designing and exploiting BP‐based catalysts and even BP‐analog‐based catalysts with wide practical application.

### 3.1 Mechanism of BP in Photo/Electrocatalysis

#### 3.1.1 The Reaction Mechanism of Photocatalysis

**Figure 4:**
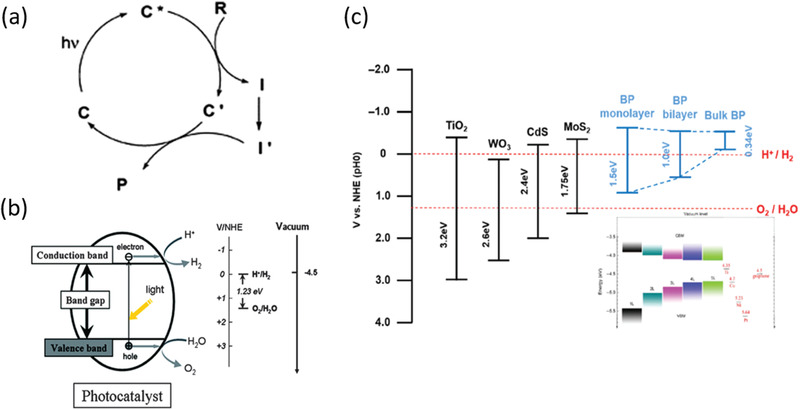
a) Schematic of reaction cycles of a photocatalyst C in reagent R to product P transformation. Reproduced with permission.^[^ ^54^ ^]^ Copyright 2000, American Physical Society. b) Illustration of thermodynamic limits of water splitting hydrogen and oxygen production. Reproduced with permission.^[^ ^55^ ^]^ Copyright 2010, American Chemical Society. c) Energy alignment of various semiconductors and different layers of black phosphorus. Reproduced with permission.^[^ ^56^ ^]^ Copyright 2009, Royal Society of Chemistry.

Since the first photocatalyst TiO_2_ has been discovered by Fujishima in 1972,^[^ ^52^ ^]^ semiconductor‐based photocatalytic technology has attracted tremendous attention due to its solar‐driven nature. Numerous studies on the applications of photocatalysis have been conducted in solar energy conversion and pollutant treatment fields.^[^ ^53^ ^]^ The key for photocatalysis is to develop a stable and efficient photocatalyst which can absorb photons from visible light as well as create electron–hole pairs to efficiently initiate redox reactions. As presented in **Figure** **4**,^[^ ^54^ ^]^ the electrons on the VB of the photocatalyst were photoexcited to its CB upon being irradiated by solar light. After photocarriers migrating to the surface active sites of photocatalyst, the photoelectrons can transfer to reagent R, triggering a reduction reaction at interface, whereas the holes can transfer to the reagent R, initiating an oxidation reaction. Actually, not all the semiconductors can spontaneously trigger photocatalytic redox reactions after absorbing photon energy larger than its intrinsic bandgap. There is another necessity for the band structure of semiconductor to guarantee the photocatalytic reaction. Considering that photocatalytic overall water splitting possesses great potential to apply in sustainable energy industry, here we take it as a significant example of photocatalysis. As shown in Figure 4b, the respective CB minimum (CBM) of the photocatalyst must lie at a more negative potential level than the reduction potential of H^+^/H_2_, and the VB maximum (VBM) must lie at a more positive level than the oxidation potential of O_2_/H_2_O. In addition, the bandgap of the photocatalyst should be higher than the thermodynamics bandgap requirement (1.23 eV) in such redox reaction.^[^ ^55^ ^]^

The band structures of several semiconductor photocatalysts as well as BP are listed in Figure 4c.^[^ ^56^ ^]^ Comparing with the traditional wide‐band semiconductor such as TiO_2_, the much narrower bandgap endows BP with superiorities on solar light absorption, while partially sacrifices its strong redox capability. Fortunately, the band structure of BP is highly dependent on the layer number as well as the lateral size. For the bulk BP, both the CBM and VBM higher than the reduction potential of H^+^/H_2_, leading to direct recombination of photocarriers in BP. However, for monolayer BP, the VBM even approaches the oxidation potential of O_2_/H_2_O owing to its VBM evolving sharply with the layer number. Although not all the studies reported the exactly same bandgap for the monolayer BP experimentally, the overall evolution tendency of band structure is almost the same. Actually, the much higher CBM of few‐layer BP endows itself with strong photoreduction capability toward HER reaction under hole scavengers, while it is still a bottleneck to induce overall water splitting for pure BP.

In addition to the critical band structure requirements, the final step of photocatalysis process almost acting the same with electrocatalysis demands for superior active sites and electrical conductivity.^[^ ^57^ ^]^ Therefore, the advantageous intrinsic properties of BP discussed in the second section make it promising to apply in both photoelectrocatalysis and electrocatalysis.

#### 3.1.2 The Reaction Mechanism of Electrocatalysis

Different from photocatalytic reaction driven by photon energy, the electrocatalytic redox reaction is directly triggered by external voltage. The main purposes of introducing electrocatalyst are to lower the reaction energy barrier and accelerate the reaction rate.^[^ ^2d,e^ ^]^ Therefore, most of the attentions in electrocatalysis are focused on the redox reaction process at the interface between catalyst and solution as well as the process of charge transfer, both of which are also meaningful in photocatalytic process. Considering that the electrocatalytic HER is one of the most promising applications in the energy conversion field and BP is widely investigated in this application, here we take it as a representative to summarize the electrocatalytic mechanism.

**Figure 5:**
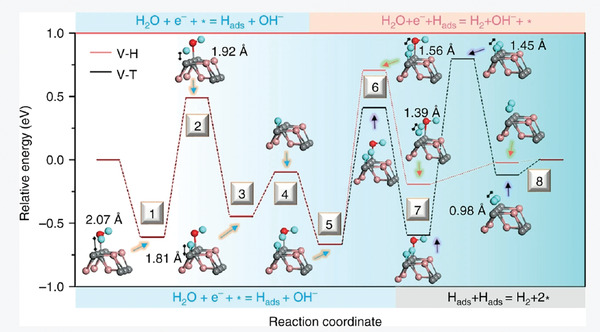
Schematic configuration‐coordinate diagrams for catalytic HER reaction mechanism. Free energy versus the reaction coordinates for Volmer–Tafel and Volmer–Heyrovsky processes (H: blue spheres; O: red spheres; pink and gray spheres correspond to FeS electrocatalyst). Reproduced under the terms of the CC‐BY Creative Commons Attribution 4.0 International license (https://creativecommons.org/licenses/by/4.0).^[^ ^2b^ ^]^ Copyright 2019, The Authors, published by Springer Nature.

Generally, there are activation, adsorption and desorption processes occurring on the reaction interfaces in the whole HER reaction as shown in **Figure** **5**.^[^ ^2b^ ^]^ First, a single H_2_O molecule couples with the electron at surface active site of electrocatalyst to form adsorbed hydrogen atom (*H*_ads_) at this active site, which is named as Volmer step. After that, there are two possible reaction pathways in the following process. One is that two *H*_ads_ at the nearby active sites directly combine with each other to form hydrogen, which is assigned as Tafel step. Another one is that *H*_ads_ at the initial active site reacts with proton from water to induce hydrogen desorption reaction, called Heyrovsky step. Accordingly, it is evident that surface active sites play the core role in the whole catalytic reduction reaction. Besides the concentration of active sites, the adsorption Gibbs energy of active sites for reactive species really makes sense for the catalytic activity.^[^ ^27a^ ^]^ In addition, the electrical conductivity decides the migration rate of charges, which is vital for the reaction rate at interface. These mechanisms correspond to the aforementioned advantageous properties of BP, reflecting its great potential in electrocatalysis as well as photocatalysis field. Nevertheless, to achieve the wide applications of BP in photo/electrocatalysis, rational regulation strategies should be adopted to overcome its intrinsic limitations and make use of its advantageous properties.

### 3.2 Regulating the Layer Number and Lateral Size of BP

Taking advantage of the evidently thickness‐dependent and size‐dependent electronic band structure, the bandgap width and conduction/valence band edge position of layered BP that are essential for solar energy absorption and redox ability of photogenerated carriers can be effectively optimized by regulating the thickness and size. Such strategy is not just meaningful for photo/photoelectrocatalysis, but also makes sense for electrocatalysis due to the electronic transport property and the proportion of surface active sites being closely bound up with layer number and specific surface area. This section focuses on the recent typical researches about the means and the mechanism of restricting the layer number and lateral dimension of BP to achieve enhanced catalytic performance.

Even though BP has already been reported the obvious superiority as cocatalyst with TiO_2_ and MoS_2_,^[^ ^7^, ^11^ ^]^ the fast photocarrier recombination arising from the narrow bandgap of 0.3 eV for bulk BP sincerely limit its photocatalytic HER performance. To conquer such challenge, the solid‐state mechanochemical ball‐milling method was adopted to acquire BP nanosheets with confined sizes of 30–60 nm and thickness of 1–8 atomic layers by Yang and co‐workers for the first time.^[^ ^12^ ^]^ As expected, the visible light photocatalytic HER rate of these nanosheets with larger bandgap of 1.21 eV reaches 18 times higher than that of bulk BP. Such dramatically enhanced photocatalytic activity is primarily attributed to the promoted photoelectron reduction ability and separation efficiency of photogenerated electron–hole pairs resulting from increased bandgap and negative shift of CB along with positive shift of VB. Meanwhile, the increased specific surface area and the introduced —OH functional groups provided by ball‐milling additive LiOH to terminate the exfoliated edge P dangling bonds contribute to the catalytic durability and activity.

**Figure 6:**
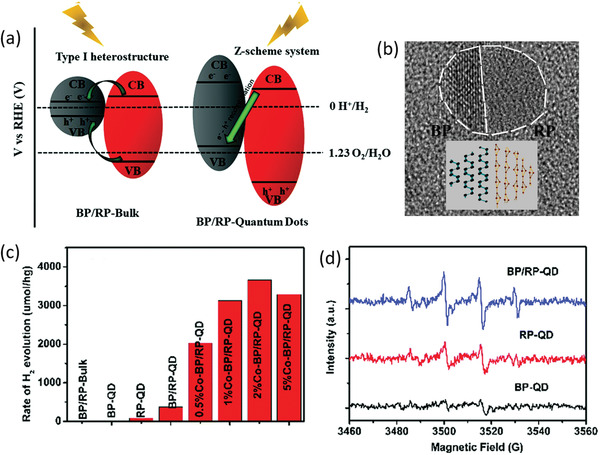
a) Schematic illustration of the energy band alignment of BP/RP‐bulk and BP/RP‐quantum dots. b) TEM image and structure schematic of BP/RP‐quantum dots. c) Photocatalytic HER rates of different samples under LED light source. d) In situ EPR results of hydroxyl radicals for various samples under visible‐light irradiation. a–d) Reproduced with permission.^[^ ^35a^ ^]^ Copyright 2019, Royal Society of Chemistry.

Through size and thickness regulation, band alignment can be achieved to construct optimal heterostructure based on BP. The recent work of Chen and co‐workers has displayed that the junction between BP and RP could be converted into Z‐scheme system from type I heterostructure through bulk BP being exfoliated into BPQDs.^[^ ^35a^ ^]^ As shown in **Figure** **6**a, both of the VB and CB positions of initial bulk BP locate in the forbidden band of RP,^[^ ^8a^ ^]^ corresponding to the type I heterostructure that photogenerated electron–hole pairs of the RP transfer to the bulk BP with weak redox ability for HER simultaneously. Such junction lacking in the charge separation of real space will lead to the fast carrier recombination and low quantum efficiency. However, for the BPQDs with 1.81 eV bandgap, the VB and CB positions undergo evidently shift and thereby the energy band with staggered alignment are constructed with RP, indicating the formation of a type II or Z‐scheme heterostructure which is much more expected to achieve charge carrier separation (Figure 6a–c).^[^ ^1^, ^58^ ^]^ Considering that the time‐resolved transient absorption spectroscopy (TAS) spectra and steady state PL spectra have demonstrated the construction of Z‐scheme system, the photoinduced free electrons in the CB of RP could diffuse to the interface and combine with the photoinduced holes in the CB of BPQDs and thereby the photoelectrons residing on the CB of BPQDs with strong reduction power could more easily migrate to the surface active sites to reduce the adsorbed species. Therefore, such BP/RP‐quantum dots exhibit much enhanced photocatalytic performance and initiate water splitting in the absence of sacrificial agents by virtue of quantum confinement effect.

Besides regulating band structure, another significant advantage of dimension control in BP is the generation of extra surface and edge active sites from the ultrathin nanosheets and super‐tiny quantum dots. Zhang and co‐workers have adopted selective centrifugation method to manifest the thickness‐dependent electrocatalytic OER performance of BP nanosheets.^[^ ^30^ ^]^ By means of reducing the thickness, the exposed surface/edge active site to adsorb oxygen species and electrocatalytic OER activity of a four‐electron transfer process have been continuously promoted. Furthermore, based on layer‐dependent property, more and more works focus on utilizing active few‐layer BP as cocatalysts. The recent reports even explore facile bottom‐up method to directly prepare BP nanosheets with various thickness via controlling solvent and temperature.^[^ ^15^, ^59^ ^]^

**Figure 7:**
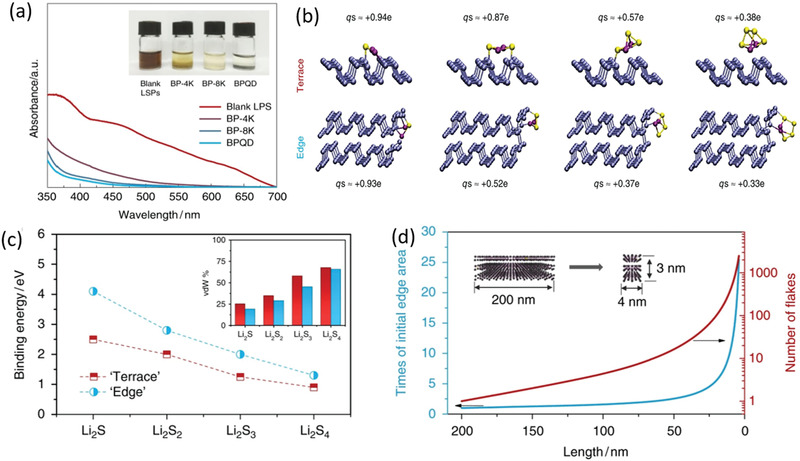
a) UV–vis spectra and photographs of LiPSs after adsorption by BP with different size. b) Snapshots of LiPSs adsorbed on planar (terrace) and edge sites respectively for monolayer and bilayer BP. qs corresponds to the partial charge on the S atom. c) The binding energies of LiSPs adsorbed on planar and edge sites of BP. The van der Waals contribution to this bond is shown in the inset. d) The evolution of exposed edge area and the number of flakes along with decreased size of BP. a–d) Reproduced under the terms of the CC‐BY Creative Commons Attribution 4.0 International license (https://creativecommons.org/licenses/by/4.0).^[^ ^35c^ ^]^ Copyright 2019, The Authors, published by Springer Nature.

Recently, by downsizing the particle size of few‐layer BP to BPQDs with numerous catalytically edge active sites and superior electrical conductivity, they exhibit sharply increased lithium polysulfide (LiPS) adsorptivity and Li_2_S precipitation capacity.^[^ ^35c^ ^]^ After loading such BPQDs, the porous carbon/sulfur cathodes exhibit rapid reaction kinetics and no shuttling of polysulfides, indicating the excellent application of BPQDs as catalysts for immobilizing and conversing polysulfide in high energy rechargeable batteries. Zhang and co‐workers adopt DFT calculations combined with experimental results to deeply reveal the mechanism of the much larger catalytic activity of BPQDs compare with few‐layer BP. According to the results of UV–vis spectra of LiPS after adsorbed by different‐sized few‐layer BP, the LiPS adsorptivities which are directly dependent on adsorbing sites display negative relation with the lateral dimension of BP (**Figure** **7**a). As the planar area difference for the few‐layer BP with the same mass is negligible, the increasingly exposed edge sites of BP should make a large contribution to the dramatically promoted adsorptivity. The DFT calculations further contrast the Li_2_S*_n_* (*n* = 1–4) adsorption kinetics respectively on planar sites with edge sites of monolayer BP with edge sites of bilayer BP as shown in Figure 7b–d. The binding energy of LiPS adsorption at the edge sites are evidently larger than at the planar sites no matter what size of the adsorbed molecule. Actually, the partial charges on Li atoms are averagely similar whether the LiPS is adsorbed at edge or planar sites, while the S‐atom part is adsorbed at edge sites with lower partial charges, indicating that the bonding is less ionic. Such stronger covalent character of bonding formed between edge sties and LiPS should result from the higher activity of undercoordinated P at the zigzag terminated edges contrast with the perfectly coordinated P at the surface. As a result, the decreasing lateral size of BP along with the increasingly exposed edge active sites will significantly contribute to the catalytic activity and redox reaction kinetics process, which provides an effective strategy to exploit the catalytic property of BP in depth.

### 3.3 Metal Incorporation

Metal incorporated into the honeycomb framework of BP or just anchored on the surface/edge planes of BP can sharply modulate the catalytic performance by efficiently accelerating carrier mobility, enhancing separation efficiency, promoting light absorption, increasing and even providing reactive sites.^[^ ^32^, ^60^ ^]^ Considering that a wide variety of metal atoms such as Co, Ni, Pt exhibit the moderate Gibbs free energy closed to zero which facilitates the adsorption and desorption of hydrogen,^[^ ^61^ ^]^ incorporating these metals onto the exposed surface of BP can really make sense to the photo/electrocatalytic HER. Furthermore, the only three‐coordinated honeycomb structure endows each layer of BP with exposed lone pair electrons which exhibits negative adsorption energy to metal atoms, indicating that BP owns superior activity to bond with metal atoms and even activate the metal catalysts.^[^ ^61b^ ^]^ Besides, plasmonic metallic nanoparticles like Au, Ag can be introduced to combine with BP to enhance solar energy absorption and steer interfacial hot electron injection,^[^ ^62^ ^]^ resulting in the higher quantum efficiency in photocatalytic field. In this section, we will focus on the recent progress about taking advantage of metal atoms to integrate the catalytic performance of BP.

**Figure 8:**
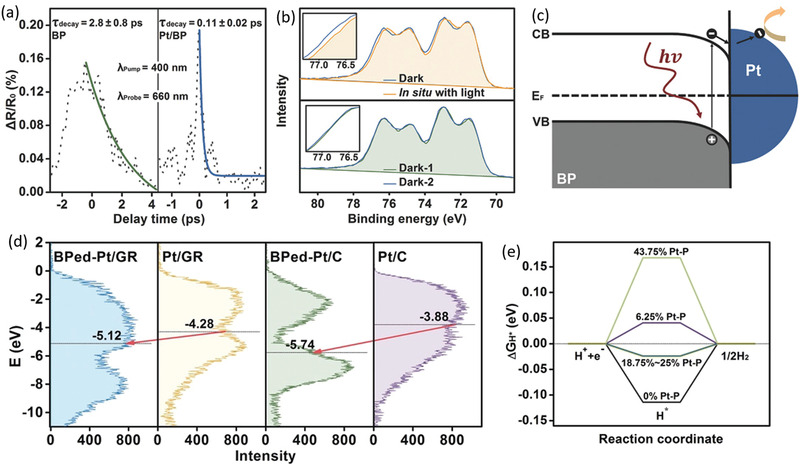
a) The ultrafast transient absorption kinetics of BP and Pt/BP probed at 660 nm. b) XPS spectra of Pt 4f core level for Pt/BP whether under 521 nm light illumination. c) Schematic of band structure and charge transfer at the interface of Pt/BP. a–c) Reproduced with permission.^[^ ^63^ ^]^ Copyright 2018, Wiley‐VCH. d) High‐resolution valence‐band XPS spectra of Pt relative to the Fermi level as an analog of the density of states with black lines indicating the d‐band centers for various samples. e) The calculated free energy of HER for various samples with different percent of Pt—P bonds. d,e) Reproduced with permission.^[^ ^61b^ ^]^ Copyright 2019, Wiley‐VCH.

To overcome the intrinsic deficiency of ultrafast charge carrier lifetime of several picoseconds for BP applying in photocatalysis, Yu and coworkers exploit the in situ growth of Pt nanoparticles on exfoliated BP nanosheets to trap photoinduced electrons of BP, inhibit the fast recombination of electron–hole pairs and thereby prolong the lifetime of excited carriers.^[^ ^63^ ^]^ As shown in **Figure** **8**a, after incorporating Pt nanoparticles and forming strong Pt–P interactions, a much shorter decay time of 0.11 ps compared with the initial 2.8 ps is observed in the ultrafast transient signal detection, indicating forming a short‐cut path for free electrons to migrate out of the BP nanosheets. To further demonstrate the transfer of photoelectrons between BP and Pt, they perform in situ X‐ray photoelectron spectroscopy (XPS) to verify the accumulation of electrons on Pt nanoparticles (Figure 8b). After being illuminated, the Pt 4f core level of Pt/BP evidently shifts by 0.2 eV to the lower binding energy, manifesting the partial reduction of Pt arising from the injection of free electrons photoinduced in BP. As illustrated in Figure 8c, the Schottky barrier forming at the interface produces a built‐in electric field which can boost the electron migrations from the CB of BP to the Pt nanoparticles, resulting in the largely enhanced photocatalytic photocatalytic efficiency even higher than commercial Pt/P25 and Pt/C.

Actually, a recent research suggests that not just Pt coordination can improve catalytic performance of BP, but also BP can act as cocatalysts to further activate Pt.^[^ ^61b^ ^]^ Utilizing the unique and negative binding energy between BP and Pt, the Pt—P bonds can be spontaneously formed and induce the strong synergistic ligand effects on Pt catalysts. It is noteworthy that such Pt—P coordination cannot be formed on another allotrope of P, red phosphorus. The XPS high‐resolution VB which is proportional to the DOS and related to the reaction species adsorption strength is used to determine the correlation between these Pt—P bonds introduced by BP and the electronic structures. The incorporation of Pt—P bonds could regulate the d‐band structure of Pt by evidently downshifting the d‐band center on the Pt surface, which are observed both in BPed‐Pt/GR and BPed‐Pt/C as shown in Figure 8d. According to the d‐band theory, as the hydrogen desorption process on BP/Pt catalysts is the rate limiting step, the downshift of the d‐band will pull more anti‐bonding states below the Fermi level, optimize the adsorption of hydrogen species and thereby sharply facilitate the HER activity. Furthermore, the DFT calculation results corroborate the introduction of 18.75–25% Pt—P bonds can lead to the increase of Gibbs free energy toward zero, which is the optimal activity for H atom being activated by surface and being desorbed without surface being poisoned (Figure 8e). Therefore, the controllable introduction of BP can effectively activate Pt for highly enhanced electrocatalytic HER. Besides Pt, BP have already been adopted as cocatalysts of other metals like Ag, Au, and successfully manipulate the electronic properties of these supported metal nanoparticles, resulting in much better catalytic ORR performance.^[^ ^64^ ^]^ Still, researchers are trying to incorporating Co onto the planar/edge sites of BP, while the spontaneous formations of CoP/Co_2_P nanoparticles on the surface impede the initial metal incorporation conceive. However, the heterostructures can be constructed between the in situ grown CoP/Co_2_P and few‐layer BP,^[^ ^42^, ^65^ ^]^ still resulting in excellent photocatalytic water splitting and electrocatalytic HER. Recently, Tian's group utilized a facile solvothermal reaction of white phosphorus and Co (NO_3_)_2_·6H_2_O in ethylenediamine to form amorphous (Co—P)‐supported BP nanosheets through free Co^2+^ spontaneously reacting with BP.^[^ ^16^ ^]^ Surprisingly, toward photocatalytic HER, such catalyst overcomes the intrinsic deficiency of slow kinetics in the four‐electron‐driven oxidation half‐reaction and conducts two‐electron process to produce ·O^2−^, ·OH, and H_2_O_2_ as hole‐oxidized products. Therefore, the photocatalytic HER from pure water was achieved via Co^2+^ incorporation. Considering that such result provides a new thought to make good use of metal atoms into BP, further explorations should be conducted to comprehend the mechanism of the voluntary two‐electron‐driven oxidation in this kind of artificial photosynthesis material.

Another typical conceive to regulate photorelated catalytic process is to introduce plasmonic metallic nanoparticles, which not only promote the spatial separation of photoinduced electron–hole pairs, but also integrate both components to coabsorb photon energy via forming plasmons and excitation.^[^ ^66^ ^]^ The localized surface plasmon resonance (LSPR) induced by metallic nanoparticles is originated from the collective excitation of conduction electrons.^[^ ^67^ ^]^ Once fulfilling the resonance conditions, such compound system can reach the maximum absorption of incident light. Based on this mechanism, Liu and co‐workers anchor Ag nanoparticles onto the BP surface and deeply investigate the effect of BP layer thickness and Ag nanoparticle size to the local field amplification and photoactivity enhancement.^[^ ^62a^ ^]^ Through decreasing the layer number of BP and increasing Ag particle size, an over 20‐fold rise is achieved in photocatalytic activity compared with pristine multilayer BP. The finite‐difference time domain (FDTD) results and photocurrent measurements indicate that the maximum field enhancements induced by LSPR are increased with Ag particle size from 20 to 40 nm and decreased with layer thickness of BP. Such electric fields are often localized at hot spots with maximized field strengths and induce the accumulation of promoted electromagnetic energy. The interaction between BP and such localized electric fields boosts the formation of electron–hole pairs with inherent short diffusion length to readily reach the BP surface and drive the photocatalytic reaction. Considering that the photoelectrons in BP can mostly relax by injecting into Ag to generate radicals with adsorbed O_2_ or transport back to BP, the resulting long‐lived holes in VB are much more active toward photocatalytic degradation. Such findings verify that the controllable metallic nanoparticle incorporation can be effective to promote the photocatalytic performance of BP, while the integration of multiple enhancement mechanism in one metal–BP system is more preferable.

### 3.4 Surface Covalent Bonding Modification

Surface covalent modification is a widely used effective strategy to introduce functional species and regulate the electronic structures for 2D semiconductor owing to the ultralarge specific surface area.^[^ ^14^, ^68^ ^]^ For few‐layer BP, covalent bonding of planar and edge sites with organic groups or molecules will not just enhance the photoelectronic or electronic properties, but also stabilize the honeycomb crystal structure and improve its durability in catalytic application.^[^ ^7^, ^12^, ^14^ ^]^ In general, considering the difficulty of introducing alien species directly in the preparation of bulk BP, it is more feasible to functionalize BP during top‐down exfoliation procedure and acquire ultrathin BP with surface covalent species simultaneously. Groups or molecules will preferentially coordinate on the exposed metastable sites with higher activation energy during BP being exfoliated and prevent these active undercoordinated P atoms from being oxidized.

**Figure 9:**
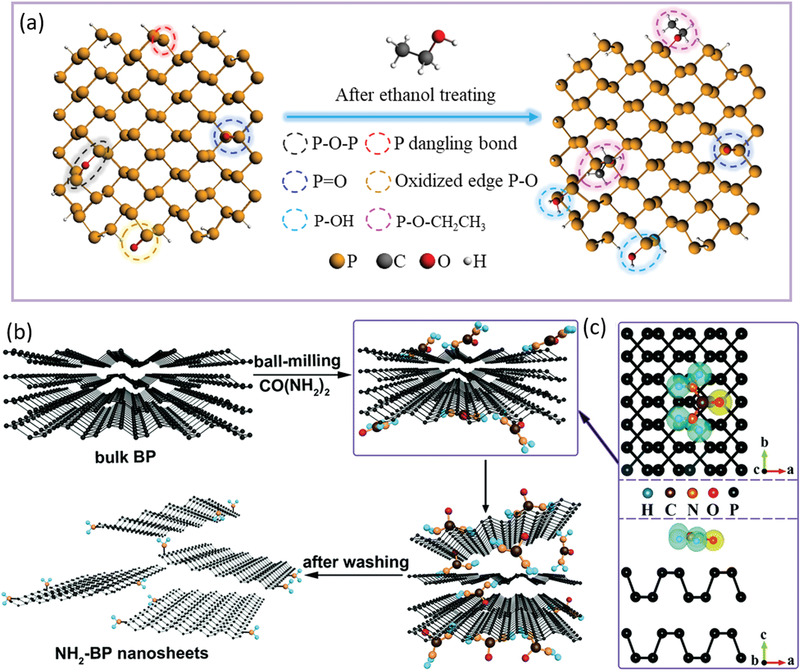
a) Top views of BPQD before and after surface modification through ethanol treatment. All the defects and surface groups are circled with different colors. Reproduced with permission.^[^ ^14b^ ^]^ Copyright 2018, Wiley‐VCH. b) Schematic of the preparation of NH_2_‐functionlized BP nanosheets with CO(NH_2_)_2_. c) Top and side views of the deformation charge density distribution for the most stable absorption configuration of CO(NH_2_)_2_ on BP. b,c) Reproduced with permission.^[^ ^14a^ ^]^ Copyright 2018, Royal Society of Chemistry.

Hydroxyl groups were first adopted to terminate the edges of BP nanosheets through ball‐milling bulk BP with LiOH additive.^[^ ^12^ ^]^ After surface functionalized with —OH groups, BP exhibits excellently recyclable visible‐light photocatalytic HER. Besides, our recent work utilized liquid exfoliation followed by solvothermal treatment in ethanol to passivate the planar and edge defects of BP into P—OH and P—O—CH_2_CH_3_ bonding structures and consequently achieved superior photoelectronic properties (**Figure** **9**a).^[^ ^14b^ ^]^ The introduction of P—OH and P—O—C bondings delocalize the absorption transition of photoelectrons between HOMO and LUMO to the central zone of BP and evidently reconstructs the favorable photoabsorption of BP, resulting in the highly efficient photon utilization and high‐quantum‐yield photoenergy conversion. Taking advantage of superior optoelectronic properties of modified BP, such method can be combined with heterostructure construction strategy to apply into photocatalysis through deriving numerous energetic electrons produced in BP.

**Figure 10:**
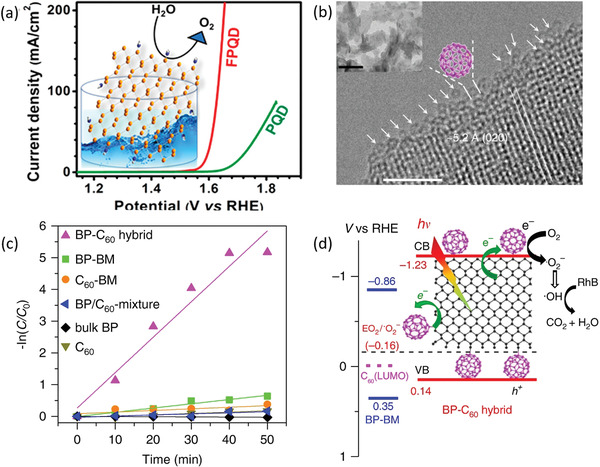
a) The linear sweep voltammetric analysis for OER activity of PQD and FPQD. The inset shows the surface‐functionalized structure of FPQD contributing to the enhanced OER performance. Reproduced with permission.^[^ ^69^ ^]^ Copyright 2018, American Chemical Society. b) High‐resolution and low‐magnification TEM images of BP‐C_60_. Nanospheres with the C_60_ molecule van der Waals diameter of 1 nm are immobilized at the edge of BP. c) Pseudo‐first‐order kinetic curves of RhB degradation for various samples. d) Schematic of the electronic band structure and photocatalytic mechanism illustration of BP‐C_60_ and BP‐BM. b–d) Reproduced under the terms of the CC‐BY Creative Commons Attribution 4.0 International license (https://creativecommons.org/licenses/by/4.0).^[^ ^71^ ^]^ Copyright 2018, The Authors, published by Springer Nature.

Besides —OH groups, amino groups have also been introduced to covalently terminate BP at the edge sites. Cheng and co‐workers reported a urea‐assisted ball‐milling method to prepare NH_2_‐functionalized BP nanosheets and achieved drastically promoted electrocatalytic HER performance with an overpotential of 290 mV at −10 mA cm^−2^ and a Tafel slope of 63 mV dec^−1^ compared those bulk BP.^[^ ^14a^ ^]^ As shown in Figure 9b,c, these small urea (CO(NH_2_)) molecules can adsorb on the surface of BP under the ball‐milling shear forces, leading to weaken the van der Waals interactions between phosphorene layers and boost the stripping effect. According to the DFT calculations focusing on the interaction between urea and BP, the adsorption energy of 0.383 eV and the only within the molecule skeleton charge density distribution suggest a typical physisorption process for urea located on the surface of BP. Meanwhile, the edges of exfoliated BP with reactive P dangling bonds can be functionalized with NH_2_ groups, which further suppress its restacking and facilitate the exfoliation. Through water washing to remove adsorbed water‐soluble urea, the NH_2_‐functionalized BP can be constructed. These NH_2_‐modified BP nanosheets exhibit dramatically higher intrinsic activity, more abundant catalytically active sites and more efficient charge transfer at the catalyst‐electrolyte, contributing to the largely enhanced electrocatalytic efficiency. Prasannachandran and co‐workers adopted another method to functionalize BP with nitrogen‐containing groups and deeply investigate the electrocatalytic promotion mechanism of such group modification.^[^ ^69^ ^]^ Although a custom‐designed electrochemical exfoliation method is used to exfoliate BP to BPQDs, choosing an appropriate electrolyte cannot just synthesis BPQDs but also lead to in situ surface functionalization in the meantime. By replacing pristine electrolyte with 0.1 wt% LiClO_4_ in formamide, the functionalized BPQDs (FPQD) are successfully prepared and exhibit much faster electron transfer kinetics once being tested as Fe^2+^/Fe^3+^ redox probe in cyclic voltammetric (CV) measurements compared to pristine BPQDs (PQD). Further, FPQD displays highly efficient electrocatalytic OER activity with an overpotential of 1.66 V at 10 mA cm^−2^ and a low Tafel slope of 48 mV dec^−1^ far better than pristine PQD as shown in **Figure** **10**a. A contrast experiment verified that FPQD with lower amount of nitrogen‐containing functional groups exhibit OER activity higher than PQD, but lower than initial FPQD. Such electrocatalytic enhancement intimately related with the surface nitrogen‐containing groups should be originated from the much higher electronegativity of N atoms than P atoms. The surface coordination of N atom can induce the electron density shift toward the nitrogen‐containing group such as —NH_2_ and leave a partial positive charge in the P atom owing to the electronegativity difference. Such charge separation on the surface can facilitate the adsorption of OH^−^ on the P atoms as proved by DFT calculations, resulting in the improved electrocatalytic OER activity.

Besides enhancing structure stability, regulating optical absorption and optimizing electrochemical activity of BP, surface covalent bonding modification can regulate band edge positions to satisfy essential factor of photocatalysis. Considering both the CB and VB position of phosphorene are more positive than the oxidation potential of O_2_/H_2_O and the reduction potential of H^+^/H_2_ which are adverse to drive water splitting, Yang and co‐workers covalently bond the edges of phosphorene nanoribbon with pseudohalogen groups (CN and OCN) to achieve desired VB/CB band edge positions which can spontaneously induce both water oxidation and hydrogen reduction without using extra energy.^[^ ^70^ ^]^ The edge electric dipole layer arising from edge modification induce the downshift of VB and CB to make the redox potentials of photocatalytic water splitting located inside the bandgap of BP, while the superior intrinsic photoelectronic properties of BP are preserved. Such modification is predicted to achieve the maximum energy conversion efficiency reaching 20% for solar water splitting cells.

Molecule covalent bonding is another effective strategy to functionalize BP. Taking advantage of the ball‐milling method, Yang and co‐workers selectively bond the C_60_ molecules at the edges of BP nanosheets to construct BP‐C_60_ (Figure 10b).^[^ ^71^ ^]^ Considering surface functionalization at the honeycomb planar sites of BP may lead to strong structure perturbation and unique electronic structure deterioration, such edge‐selective modification should be more preferable than nonselective chemical functionalization. The formation mechanism of such BP‐C_60_ is based on the generation of reactive species at the edges via a mechanochemical cleavage of P—P bonding during the exfoliation of bulk BP with high‐energy ball‐milling process. Meanwhile, the C_60_ molecules activated by high‐energy ball‐milling terminate the reactive edge sites via forming P—C covalent bonds, resulting in the edge‐modified BP nanosheets. As shown in Figure 10c, such BP‐C_60_ exhibits dramatically promoted photocatalytic activity compared with the BP/C_60_‐mixture and BP‐BM (ball‐milling BP with LiOH as additive). Such enhancement not just arises from the bonding of stable C_60_ at the edges providing a sacrificial shield which prevents BP from light, oxygen, and water attacking, but also because the introduction of C_60_ leads to a rapid photoelectron transfer from BP to the edge C_60_ and inhibits the charge recombination (Figure 10d). Inspired by these above successful catalysis promotion results, ball‐milling with an appropriate additive should be a preferable method to achieve covalent modification of BP and even can selectively functionalize their reactive edge sites rather than the susceptive planar sites.

### 3.5 Designing and Constructing Heterojunction

Achieving interface manipulation through constructing hybrid system is a strategy already widely used in catalytic field.^[^ ^32^, ^58^, ^72^ ^]^ Such method cannot just integrate the optical absorption and active sites of adjacent catalysts, but also retard the recombination of electron–hole pairs, elongate the lifetime of energetic carriers to undergo the redox reaction and thereby reduce the dissipation of input energy in the form of heat or photoemission. For 2D materials, the highly exposed surface area and ultrashort carrier diffusion distance make them more feasible to form enough heterointerface contact with other semiconductors and achieve interfacial charge migration. Considering that BP is a typical layered 2D material, it is indeed an effective way to enhance the catalytic performance of BP via heterostructure constructing with a well‐matched semiconductor or metal‐phase 2D materials. Furthermore, the highly adjustable Fermi level and CB/VB positions of BP through layer and size regulation results in the relatively controllable band alignment and electronic coupling with another material, both of which are critical for the charge transfer mechanism and quantum efficiency of the composite system. Besides, a vital advantage for BP constructing hybrid system is to cover itself with chemically stable materials and protect itself from degradation.^[^ ^27a^ ^]^ Therefore, numerous reports have been focused on figuring out well‐matched couple materials and ideal preparation techniques to construct heterostructure with BP and sharply facilitate its catalytic activity and durability. In this section, we will conclude the recent work about interface manipulation in photocatalysis and electrocatalysis of BP and comprehend the mechanism of huge enhancement.

#### 3.5.1 Effective Interfacial Coupling and Rational Band Alignment for Promoted Photocatalysis

Toward photocatalysis, the core of heterojunction assembly is to achieve enhanced light harvest and efficient charge separation to respectively produce enough photogenerated charge pairs and guarantee the carrier migration to surface active sites. Generally, according to the relative positions of CBs/VBs between two coupled semiconductors and the actual charge flow directions, the formed binary heterostructures can be mostly divided into type I, type II, and the special Z‐scheme.^[^ ^1^, ^73^ ^]^ Among them, type II and Z‐scheme systems are more favorable for photocatalytic reaction owing to the much more effective separation of photoinduced negative and positive carriers in real space.^[^ ^58^, ^74^ ^]^ Thus, during designing heterojunction for BP, in addition to the intrinsic photoelectronic properties and catalytic activity of the couple semiconductor, an optimized band alignment should also be taken into consideration.

**Figure 11:**
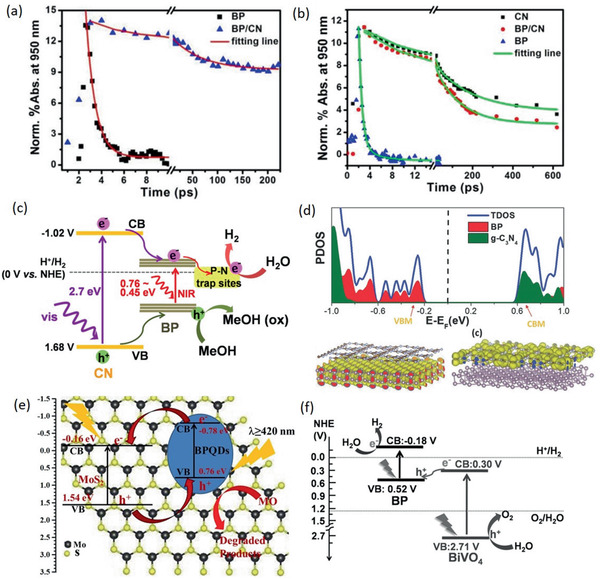
a,b) The normalized kinetics of TDR spectra probed at 950 nm for various samples under 780 nm light excitation (a) and 400 nm light excitation (b). c) Mechanism schematic of the visible and NIR light photocatalytic HER for BP/CN in the presence of methanol. a–c) Reproduced with permission.^[^ ^32b^ ^]^ Copyright 2017, American Chemical Society. d) Calculated band structure and charge distribution of the VB maximum (VBM) and CB minimum (CBM) for BPQD‐CN heterostructure. Reproduced with permission.^[^ ^35b^ ^]^ Copyright 2018, Wiley‐VCH. e) Mechanism schematic of photocatalytic MO degradation for BPQDs/MoS_2_ type II heterostructure. Reproduced with permission.^[^ ^46^ ^]^ Copyright 2018, Elsevier B.V. f) Mechanism schematic of photocatalytic overall water splitting for BP/BiVO_4_ Z‐scheme system under visible light irradiation. Reproduced with permission.^[^ ^47b^ ^]^ Copyright 2018, Wiley‐VCH.

To successfully guarantee interface charge transfer, the large fraction of heterointerface contacts and strong interfacial interaction between BP and the couple semiconductor are indeed essential, indicating the preference of constructing 2D/2D or 2D/0D heterostructure with sufficient contact area. Thus, as another layered 2D materials with wider bandgap, g‐C_3_N_4_ (CN) is promising to form binary nanohybrid with BP and construct the metal‐free photocatalyst with wide range light harvest from UV to near‐infrared (NIR). Wang and co‐workers designed a hybrid system composed of BP and CN nanosheets (BP/CN) with highly improved photocatalytic HER performance under the irradiation of wavelength >420 and >780 nm for the first time.^[^ ^32b^ ^]^ As shown in **Figure** **11**a,b, taking advantage of the time‐resolved diffuse reflectance (TDR) spectroscopic measurements under 400 and 780 nm light irradiation, which respectively correspond to excite both semiconductors and only BP, the kinetic mechanism of photogenerated charge transfer is deeply unraveled. Under NIR light excitation, the only exited BP will induce photoelectrons and the P–N coordinate bond at the interface will trap these excited electrons, leading to the evidently prolonged lifetime for the BP/CN composites compared to the BP and boost the photoelectrons to effectively reduce water molecules at the trap sites instead of rapid recombination with holes. Under visible light excitation, the shorter lifetime for BP/CN composites is due to the photoelectron transfer from excited CN to adjacent BP and BP act as electron acceptor to further produce H_2_, consistent with type I heterojunction between the BP and CN nanosheets (Figure 11c). As a result, inhibiting the fast charge recombination via the strong interfacial interaction and efficient electron migration at the interface drastically improves its photocatalytic HER performance. Similar to such strategy, Yu and co‐workers recently adopted a simple ball‐milling method to acquire BP/CN heterostructure with interfacial P—N bonds from economical urea and red phosphorus (RP) and achieve excellent photocatalytic HER and rhodamine B (RhB) degradation activity.^[^ ^75^ ^]^ Based on the cheap raw materials and facile method, it is promising to synthesis such efficient metal‐free photocatalysts on a large scale.

Further investigations focused on 2D BP/CN nanohybrids with different weight ratios of two components have been conducted by Wang's group and Qiao's group with two different synthesis methods.^[^ ^76^ ^]^ In addition to the highly promoted visible‐light photocatalytic activity at an optimal weight ratio, both investigations manifest the evident type I heterojunction between BP and CN nanosheets, consistent with the abovementioned results. Besides, the DFT calculations indicate the higher Fermi level of CN, which can induce the electron migration from CN to BP after they contact to form junction.^[^ ^76b^ ^]^ Such constructed strong interfacial electronic coupling facilitates the proton reduction at the BP surface active sites. According to the recent work, this kind of 2D/2D BP/CN nanohybrids display a wide range of photocatalytic applications including molecular oxygen activation, nitrogen fixation, and bacterial inactivation owing to the increased photoabsorption and carrier separation efficiency.^[^ ^76^, ^77^ ^]^ These results not only provide a highly potential heterostructure photocatalyst worth being further explored in practice, but also figure out the sharply enhanced mechanism which can be applied in the area of energy conversion.

Actually, as mentioned above, the type I heterojunction is not the most ideal one to satisfy the efficient spatial charge separation. Size reduction to acquire the 0D BPQDs from the 2D BP can convert the initial type I band alignment with CN nanosheets into the type II.^[^ ^78^ ^]^ Recently, Yan and co‐workers loaded BPQDs onto the layered CN to assembly a 0D/2D heterostructure through high‐vacuum stirring method.^[^ ^35b^ ^]^ The BPQD‐CN nanohybrids shows superior photocatalytic water splitting performance whether under simulated sunlight or the monochromatic LED light irradiation. Actually, the CB and VB edge positions (vs NHE) of BPQDs respectively exhibit negative shift and positive shift compared with BP nanosheets, leading to the well‐matched energy band with staggered alignment between BPQDs and CN. As corroborated by the charge distribution in the real space according to the DFT calculations, the VB and CB of heterojunction are mainly contributed by BP and CN (Figure 11d). The built‐in electric field of such type II heterojunction induced by the diffusion of charges will drag the photoinduced electrons and holes separately onto CN and BP, thereby resulting in efficient photocarrier separation in the real space and inhibiting the recombination of free carriers. Besides, the high hole mobility of BP further facilitates the hole receiving and migration. These results indicate that the highly enhanced charge separation due to the strong electronic coupling in such type II heterojunction can largely contribute to the superior photocatalytic activity. In addition to CN, RP and MoS_2_ can also architect this kind of staggered band alignment with BPQDs.^[^ ^35^, ^46^ ^]^ Liu's group adopted economical grinding and sonicating approach to anchor BPQDs onto the MoS_2_ nanosheets and form 0D/2D heterojunction with type II band alignment.^[^ ^46^ ^]^ Such nanocomposites exhibit dramatically enhanced photocatalytic activity toward methylene orange (MO) degradation whether under visible‐ and NIR‐irradiation. The sharply increased photogenerated electron–hole separation for the BPQDs/MoS_2_ heterostructure is demonstrated by the steady‐state PL and ultrafast TA results. Such efficient suppression of charge recombination is arising from the reverse spatial migration of photoexcited electron and holes, which is guaranteed by the more negative CB and VB band edges of BPQDs than MoS_2_ (Figure 11e). Furthermore, the formation of such type II heterojunction with effective photoexcited charge transfer is contributed by the interfacial bonding P–S and P–Mo, as proved by the overall results of XPS, X‐ray absorption near‐edge structure (XANES), and nuclear magnetic resonance (NMR). Actually, these results indicate that an optimal band alignment should be combined with the effective interfacial electronic coupling to construct the appealing heterojunction with superior photocatalytic quantum efficiency, while BP with numerous uncoordinated lone pairs is more conducive to form such kinds of interfacial interactions with the adjacent semiconductor. In other words, architecting heterojunction to enhance photocatalysis is definitely an appropriate strategy for BP due to such relatively high surface activity and tunable band structures.

Generally, compared with the above heterostructures, Z‐scheme system which simulates the natural photosynthetic mechanism in the chloroplast is more favor of achieving environment‐friendly photocatalytic energy conversion without sacrificial agents.^[^ ^1^, ^74^, ^79^ ^]^ Considering it can be constructed with two narrow‐bandgap semiconductors with staggered‐alignment energy bands and separately utilize the higher CB and lower VB of both semiconductors to conduct redox reaction, the Z‐scheme system can not only harvest broadband light even from visible to NIR, but also provide large overpotentials and strong redox capability whether for reduction or oxidation half‐reaction in overall photocatalysis. Recently, taking advantage of electrostatic interactions, Majima and co‐workers assembled BiVO_4_ nanosheets onto the surface of BP nanosheets and constructed 2D/2D nanohybrids with overall photocatalytic pure‐water splitting under light irradiation large than 420 nm.^[^ ^47b^ ^]^ Via XPS analysis, the sufficient interfacial interactions which are essential for the interfacial charge transfer were corroborated in such hybridized system by evident binding energy shifts and electron density variations. According to the UV–vis diffuse reflectance spectra and Mott–Schottky plots; both the CB and VB levels of BP are more negative than BiVO_4_ as displayed in Figure 11. Different from kinetics in type II heterojunction, the photoexcited electrons on the CB of BiVO_4_ can immediately recombine with the photogenerated holes on the VB of BP under visible‐light irradiation due to their close band positions, presenting the photoelectron kinetics of ideal Z‐scheme system as displayed in Figure 9e.^[^ ^74a^ ^]^ Therefore, the reserving excited electrons on the CB level of BP which is negative than the reduction potential of protons can effectively conduct photocatalytic HER and in the meanwhile, the leaving reactive holes on the VB of BiVO_4_ which is positive than the oxidation potential of water can be utilized to undergo OER reaction. The successful construction of such Z‐scheme BP/BiVO_4_ was demonstrated by the respectively shorter and longer lifetime of photogenerated electrons and holes in BiVO_4_ through TAS spectra. The elongated excited carrier life time induced by prominently spatial charge separation combined with the totally crossed redox potential result in the overall photocatalytic water splitting without sacrificial agents and external bias. Similarly, Lu and co‐workers utilized CTAB‐assisted self‐assembly method to decorate Bi_2_WO_6_ nanosheets with sandwich structures onto the surface of BP nanoflakes to construct Z‐scheme heterojunction.^[^ ^32a^ ^]^ Such 2D/2D nanocomposites not only exhibit highly enhanced photocatalytic water splitting efficiency, but also excellent capability in the photocatalytic NO removal. The promoted photoactivity of BP for applying in the pollutant treatment area highly relies on the interface synergy effect. These results indicate that such kind of layered perovskite is conducive to architect Z‐scheme system with BP nanosheets owing to the well‐matched band structures. Besides, via a rational energy band regulation, the BP can form Z‐scheme heterostructure with other more photoactive semiconductors such as the abovementioned BP/RP.

#### 3.5.2 Steering Directional Electron Injection for Promoted Electrocatalysis

Toward electrocatalysis reactions, lacking photoenergy participation makes it more conducive to focus on the adsorption and desorption procedures at the surface‐active sites of catalysts, which are quite essential for any kind of catalytic reaction.^[^ ^2^, ^57^, ^80^ ^]^ The ideal active sites with moderate Gibbs free energy (Δ*G*) is crucial for adsorbing species and desorbing the products.^[^ ^2^, ^57^ ^]^ Actually, in addition to the number of active sites, the intrinsic exchange current density (*j*_0_) also plays a significant role for catalytic activity, which is partially indicated by the volcano‐type relationship between *j*_0_ and Δ*G* in the catalytic HER field.^[^ ^81^ ^]^ Constructing hybridized heterostructure with directionally interfacial charge transfer can optimize the electron density and even current density. Based on this conceive, Zeng and co‐workers deposited catalytically active MoS_2_ nanosheets onto BP nanosheets to architect 2D/2D heterointerfaces and achieve the highly promoted electrochemical HER performance with the overpotential as low as 85 mV at 10 mA cm^−2^.^[^ ^82^ ^]^ The higher Fermi level of BP compared with MoS_2_ result in the electron migration from BP to MoS_2_ and accumulation onto the MoS_2_ in such nanohybrids. The injected electrons from BP facilitate the interaction of electrons with protons at electrolyzed interface and induce the 22‐time‐higher *j*_0_ than pure MoS_2_, leading to the much‐enhanced catalytic activity of the active sites on the surface of MoS_2_. As proved in DFT calculations, these results are consistent with the decreased Δ*G* and increased adsorption of the active sites after electron injection. These results provide an effective strategy of heterointerface design to engineer the electronic characteristics and regulate the activity of active sites rather than increasing the active‐site‐number or active‐surface‐area.

**Figure 12:**
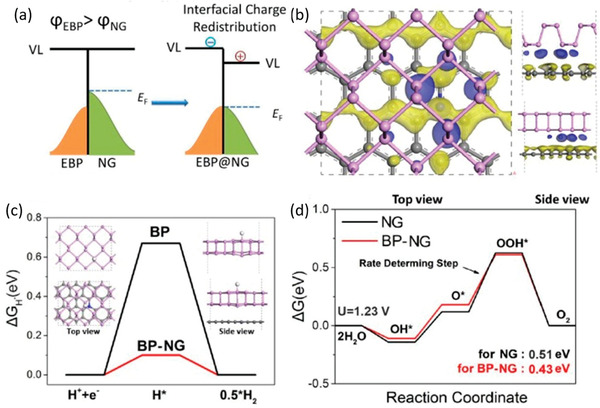
a) Schematic of the charge redistribution at the interface of CBP@NG steered by Fermi level difference. b) Illustration of the differential charge density (blue and yellow separately for electron‐rich area and hole‐rich are). c) The calculated free energy diagrams of HER for BP and BP‐NG. d) The calculated free energy diagrams of OER process for NG and BP‐NG. a–c) Reproduced with permission.^[^ ^27a^ ^]^ Copyright 2019, American Chemical Society.

Recently, the integrated electronic configuration to dually optimize the electronic structures of each component of BP‐based heterostructure has been achieved through the similar directional interfacial charge transfer. Yu and co‐workers exploited the electrostatic interaction between negatively charged ultrathin exfoliated BP (EBP) and positively charged N‐doped graphene (NG) to construct the metal‐free 2D/2D heterostructure EBP@NG and accomplished impressive overall water splitting with excellent durability due to its bifunctional activities toward both catalytic HER and OER.^[^ ^27a^ ^]^ Such superior dual activities are contributed by the rational heterointerface engineering. Besides laying a solid foundation to the interfacial electronic coupling between EBP and NG, the large‐area face‐to‐face intimate contact in the 2D/2D heterostructure also effectively passivates the surface P atoms and protects EBP from structure deterioration. Furthermore, the core of such heterointerface design is the lower Fermi level of EBP compared to NG, which will be energetically favorable for the electron injection from NG to EBP and hence induce the interfacial charge redistribution as presented in **Figure** **12**a. According to the calculated differential charge density distribution in real space, the EBP attracts electrons from the adjacent NG and architects the electron‐rich EBP surface and the hole accumulated NG surface in the hybrid system (Figure 12b). Toward HER half‐reaction, the increased electron density of EBP effectively reduces the free Gibbs energy of hydrogen adsorption (Δ*G*_H_ from 0.67 to 0.1 eV) on the active sites at EBP surface and leads to a better stabilization of adatom H, thereby dramatically boosting its HER activity (Figure 12c). In the meantime, toward the OER half‐reaction, the activated positive‐charge carbon sites at the surface of NG will exhibit lower Gibbs free energy barrier of forming OOH* species (*G*_O* → OOH*_ = 0.43 eV) than the initial 0.51 eV and be more favor of the conversion from O* to OOH* which is regarded as the key rate‐determining step of OER (Figure 12d). As a result, such a directional interfacial electron injection strategy simultaneously modulates the electronic structures of both components and promotes the catalytic HER and OER activities, leading to the excellent electrocatalytic overall water splitting for EBP@NG heterostructure. These results not only dig into the electronic coupling mechanism of catalytic kinetic process but also pave a way for designing BP‐based catalysts with bi‐functional activities instead of neglecting one of the components in the interfacial electronic engineering.

#### 3.5.3 Multifunctional Ternary Heterostructure Construction for Promoted Photo/Electrocatalysis

Although the abovementioned binary heterojunction construction of BP has successfully introduced highly enhanced photo/electrocatalytic capabilities and wider applications including the highly desirable overall water‐splitting, NO removal, and CO_2_ reduction, the quantum efficiency for most of them is still far from commercial practices. Furthermore, the inadequate interfacial contact and electronic coupling in some binary hybrid system such as certain 2D/3D heterostructure make it necessary to introduce a mediator between them.^[^ ^48^, ^83^ ^]^ In most situations, metal atoms with superior electron conductivity are the optimum selection. Actually, the incorporation of metal into the binary system may induce multiple advantages such as plasmonic enhancement or increased active sites. In this section, we will explore the recent researches focusing on BP‐based ternary heterojunction consisting of BP, semiconductor, and metal.

Recently, Majima's group incorporated plasmonic metal, Au nanoparticles, into 2D BP and 2D La_2_Ti_2_O_7_ (LTO) hybrid system and architected BP‐Au/LTO ternary nanohybrids with 74‐fold enhancement of photocatalytic HER efficiency compared to pure BP.^[^ ^84^ ^]^ In such nanohybrid system, the superior broad light absorption from UV to NIR is not merely contributed by BP, but also arising from the wide surface plasmon resonance (SPR) absorption centered at 536 nm of Au. More importantly, the introduction of Au determinably converses the charge migration in the composites. The SPR excited hot electrons overcome the interfacial Schottky barrier of Au and LTO and migrate to the CB of LTO to reduce H^+^. Under the strong interaction of BP and Au, the excited electrons of BP effectively transfer to the electron‐deficient electronic state of Au. The introduction of plasmonic Au induces the efficient charge transfer from BP and Au to LTO and thereby sharply enhances the photocatalytic activity. More recently, a similar conceive was adopted to design a ternary heterostructure consisting of BPQDs, Au nanorods and CdS nanowires by Zhang's group.^[^ ^48^ ^]^ Even though CdS inherently possesses typical type II band alignment with BPQDs, the unmatched work function between BPQDs and CdS may lead to a large Schottky barrier and inhibit the charge‐transfer from BP to CdS at the interface. Thus, it is imperative to introduce Au as the mediator for facilitating an Ohmic contact and decreasing the Schottky barrier. According to their first‐principle calculations, once the Au contacts with CdS, the higher work function of Au will induce the Schottky barrier, which is favorable for the migration of SPR hot electrons from Au to CdS as well as suppressing the backflow of injected hot electrons from CdS to Au. Besides, the matched work function between Au and BPQDs induces a well‐established Ohmic contact with negligible contact barrier. Different from the low‐positioned CB edge of BP nanosheets in the above BP/Au/LTO system, the CB in such BPQDs exhibits evidently up‐shift and higher than the longitudinal surface plasmon resonance (LSPR) energy level of Au nanorods with LSPR peak at 860 nm, hence leading to the excited electrons on the CB of BPQDs effectively transfer to Au without contact barrier. In the meantime, the hot electrons are generated in Au through SPR excitation. All these energetic electrons can unidirectionally migrate to CB of CdS and conduct reduction reaction, resulting in the outstanding photocatalytic HER capability. These results further demonstrate that certain plasmonic metal can be deliberately introduced to become electron relay and SPR electron donor between BP and another semiconductor to overcome their unmatched work function and insufficient interfacial adhesion and boost strong absorption from UV to NIR regions. Through rational band alignment and work function design, an effectively unidirectional energetic electron migration can be achieved in BP‐based ternary heterostructure for the high‐quantum‐efficiency photocatalysis.

In addition to the above kind of plasmonic‐metal‐regulated ternary photocatalysts, another kind of BP‐based ternary heterostructure toward promoted electrocatalysis capability has been reported recently. Min and co‐workers anchored Pd nanoparticles onto the titanium dioxide nanosheets/BP (ATN‐BP) hybrids and exploited a ternary electrocatalyst with excellent ethanol oxidation reaction (EOR) performance.^[^ ^85^ ^]^ In such system, Pd metal nanoparticles exhibiting relatively high catalytic activity for ethanol oxidation selectively immobilize and grow on the boundaries between the ATN and BP nanosheets, leading to the formation of interface contacts among three components. Besides the intercalated‐structure promoted electrolyte penetration, another essential element for catalytic enhancement is the formation of P—O—Ti bonds between BP and ATN, which facilitates the electron migration from BP to interconnected ATN. After incorporating Pd as catalytic active sites, the effective electron transfer from ATN/BP to Pd induces the sharply increased charge density of Pd, thereby leading to the excellent catalytic EOR performance. The superior carrier mobility of BP is further conducive to the whole electron transport procedure.^[^ ^85^ ^]^ As active oxygen species can participate in the catalytic oxidation of the intermediates adsorbed on the Pd sites, the excellent water adsorption capacity of ATN/BP inducing increased concentration of the OH— species at the interface can lead to the accelerated removal of these reactive intermediates and regeneration of the Pd‐free active sites. These results further corroborate that the integration of superior electron migration property and catalytic active sites through synergistic interaction of ternary heterostructure especially interfacial interaction with suitable metal can achieve drastically enhanced catalytic performance.

## 4 Applications of BP in Catalysis

### 4.1 Application of BP as a Photocatalyst

Semiconductor‐based photocatalytic technology has attracted huge attention since the realization of photocatalytic water splitting on TiO_2_ electrode.^[^ ^20^ ^]^ In the beginning, similar metal oxide semiconductors such as ZnO, In_2_O_3_, CuO with wide bandgap have been deeply digged in this field.^[^ ^83^, ^86^ ^]^ However, a common fatal drawback of these metal oxides is the light harvesting almost restricted in only UV band. Even though numerous efforts have been devoted to broaden their photon absorption to the blue band through regulation strategies such as introducing intrinsic defects for narrowing the optical bandgap, the solar energy conversion efficiency still remains poor.^[^ ^20^ ^]^ Considering the inevitable limitation of metal oxides, the transition‐metal‐dichalcogenides such as CdS with narrower bandgap evoke research interests, while the high charge recombination rate and instability in acidic/alkaline solution still limit their application. Actually, another critical drawback of these traditional metal‐based photocatalysts is the toxic and corrosive nature as well as the relatively high cost compared with those metal‐free semiconductors in future commercial application.^[^ ^32c^ ^]^ Accordingly, as a metal‐free 2D semiconductor, g‐C_3_N_4_ has been investigated and regulated for years for the purpose of achieving superior performance in photocatalytic applications.^[^ ^87^ ^]^ Nevertheless, its photocatalytic activity is restricted by lacking sufficient and efficient active sites as well as light harvesting in long wavelength band. In the contrast, BP is also a layered 2D semiconductor consisting of earth‐abundant element with low cost, while it overcomes the aforementioned limitations on catalytic activity, solar absorption, and conductivity.

Although the photocatalytic application of BP had been expected theoretically before 2016, there were only a few reports corroborating its photocatalytic ability during that period of time. The initial studies about its photocatalytic application mainly focused on organic degradation.^[^ ^8^, ^62^ ^]^ In 2017, Yang's report was a main breakthrough which indicated that pure BP could achieve superior photocatalytic HER activity.^[^ ^12^ ^]^ The reported HER rate was comparable or even higher than g‐C_3_N_4_. In 2018, Majima's group reported photocatalytic full water‐splitting in the 2D Z‐scheme heterostructure consisting of BP and BiVO_4_, which is a rarely reported phenomenon in those traditional photocatalysts.^[^ ^47b^ ^]^ More recently, Tian's group reported amorphous cobalt phosphide supported BP nanosheets achieve a state‐of‐the‐art apparent quantum efficiency (AQE) of 42.55% at 430 nm and solar‐to‐hydrogen (STH) of over 5.4% for photocatalytic HER from pure water without any hole scavenger.^[^ ^16^ ^]^ The photoinduced holes are found to produce ·O_2_^−^, ·OH, and H_2_O_2_ instead of O_2_, which unravels a newly possible reaction mechanism. These breakthroughs for BP in photocatalysis field evolving with time reflects its future potential in solar energy conversion and pollutant degradation fields. A brief summary of BP‐based photocatalysts for photocatalytic applications has been shown in **Table** **1**. In the following sections, we will introduce its applications in these two fields more extensively.

**Table 1:** Summary of BP‐based photocatalysts for photocatalytic applications (*N* represents number)

Photocatalytic applications	Samples	Cocatalyst	Light source	H_2_ production [µmol g^−1^ h^−1^]	O_2_ production [µmol g^−1^ h^−1^]	RhB degradation rate [min^−1^]	Ref.
Water splitting	BP nanosheets	N	*λ* > 420 nm	64			^[^ ^89^ ^]^
	BP/RGO	Pt	*λ* > 420 nm	3400			^[^ ^90^ ^]^
	BP‐BM	N	*λ* > 420 nm	512			^[^ ^12^ ^]^
	BP/g‐C_3_N_4_	N	*λ* > 420 nm	427			^[^ ^32b^ ^]^
	(BPQDs)/CN	Pt	*λ* > 420 nm	271			^[^ ^93^ ^]^
	BP/CdS	CS	*λ* > 420 nm	11 192			^[^ ^94^ ^]^
	BP/BiVO_4_	N	*λ* > 420 nm	160	102		^[^ ^47b^ ^]^
	BP/RP	N	*λ* > 420 nm	330			^[^ ^97^ ^]^
	BP/RP	Co	*λ* > 420 nm	2960			^[^ ^97^ ^]^
Dye degradation	BPQDs	N	*λ* > 420 nm			0.81	^[^ ^107^ ^]^
	BP/CN	N	*λ* > 420 nm			0.288	^[^ ^76a^ ^]^
	BP/RP	N	*λ* > 420 nm			0.069	^[^ ^8a^ ^]^
	BP/Ag	N	*λ* > 420 nm			0.0574	^[^ ^62a^ ^]^

#### 4.1.1 BP for Water Splitting

**Figure 13:**
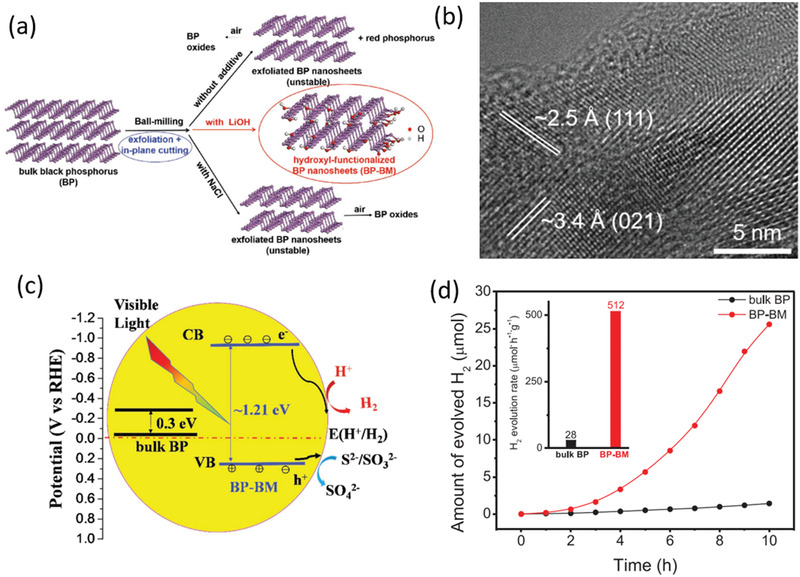
a) Scheme of the ball‐milling treatment of BP with or without additive. b) HR‐TEM image of BP‐BM. c) the mechanism of the photocatalytic H_2_ evolutions of BP‐BM and bulk BP. d) H_2_ evolution rates of bulk BP and BP‐BM. a–d) Reproduced with permission.^[^ ^12^ ^]^ Copyright 2017, Wiley‐VCH.

As the bandgap width and conduction/valence edge position of BP play an important role for solar energy absorption and redox ability, modulating the BP's band structure really make sense for photocatalysis. Owing to its thickness‐dependent band gap structure, the bandgap width can be facilely regulated by layer numbers. Besides, the bandgap of BP can be further tuned via tensile strains, and the band edge alignments can be adjusted at certain pH.^[^ ^88^ ^]^ The bandgap of phosphorene was tuned from 1.54 eV at ambient condition to 1.79 eV under 5% tensile strains along *a* axis and 1.82 eV under 7% tensile strains along *b* axis. The tuned bandgap contributes to an enhanced absorption ability of BP in visible light range, leading to a higher efficiency conversion of solar energy. At ambient condition, BP is not suitable for water splitting because the VBM of BP is more negative than the redox potential of O_2_/H_2_O. However, the redox potential for water splitting reaction can be shifted upward 0.472 V in pH = 8.0 condition, thus the VBM of BP lies appropriately positive than the redox potential of O_2_/H_2_O. The evolution of hydrogen from water by BP nanosheets under visible light illumination was then experimentally demonstrated in 2017, even though the BP nanosheets exhibited weak photocatalytic activity (with only 64 µmol H_2_ production by 45 µmol BP in 1 h).^[^ ^89^ ^]^ To improve the H_2_ evolution efficiency, the cocatalyst Pt was used due to its low overpotential for proton reduction to produce H_2._ Although the photocatalytic activity was improved to some extent, the H_2_ evolution activity is still poor due to the limited electrons transfer between BP and Pt. By introducing electron mediators may be an effective way to solve this problem. Zhu et al.^[^ ^90^ ^]^ have employed 2D reduced graphene oxide (RGO) as an electron mediator for BP and Pt. When BP is photoexcited, the electrons generated on the BP can first transfer to the surface of RGO and then effectively move to Pt surface. The RGO act as a bridge between BP and Pt for the enhanced the electrons migration. With enough electrons accumulated on Pt, the H_2_ evolution is induced. The hybrids exhibit an enhanced photocatalytic activity for H_2_ evolution, as the optimum H_2_ evolution rate is 3.4 and 0.84 mmol g^−1^ h^−1^ under >420 and >780 nm irradiation for 4 h, respectively. Considering the toxic, corrosive, and expensive nature for metal‐based cocatalyst systems, a metal‐free system based on BP nanosheets was proposed for H_2_ evolution.^[^ ^12^ ^]^ The BP nanosheets were synthesized from ball‐milling bulk BP (BP‐BM) in the presence of LiOH. As shown in **Figure** **13**, hydroxyl groups (—OH) were terminated on the exfoliated P dangling bonds, which inhibited the surface degradation and enhanced stability of BP nanosheets. Once being exfoliated into few layer nanosheets, the bandgap of BP‐BM increased from 0.3 to 1.21 eV, which will effectively inhibit the undesirable electron–hole recombination. The hydrogen evolution rate of BP nanosheets reached 512 µmol h^−1^ g^−1^, which is about 18 times higher than that of bulk BP and is comparable to that of g‐C_3_N_4_.

**Figure 14:**
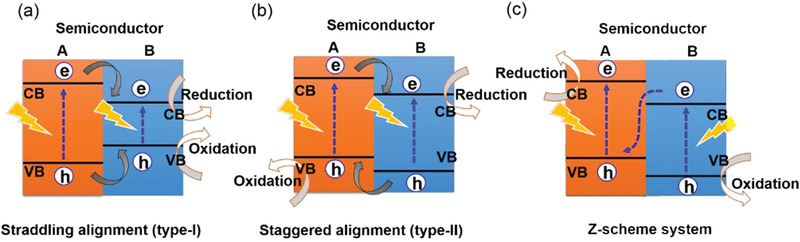
Schematic illustration of the three different types of semiconductor heterojunction photocatalysts: a) type I, b) type II, and c) Z‐Scheme system. a–c) Reproduced with permission.^[^ ^91^ ^]^ Copyright 2019, Wiley‐VCH.

Another problem for BP's poor photocatalytic activity is the rapid recombination rate of photogenerated carriers. The construction of BP‐based heterostructure has been utilized to realize the separation as the electrons or holes will transport to different catalysts surfaces to avoid the recombination. According to their band alignments, the BP‐based semiconductor heterojunctions for photocatalytic water splitting can be divided into three types: types I and II heterojunctions and Z‐scheme systems (**Figure** **14**).^[^ ^91^ ^]^ Toward type I heterojunction, the CB and VB positions of semiconductor A are respectively higher and lower than the corresponding band positions of semiconductor B. As a result, the photoexcited electrons on the CB of semiconductor A will migrate to the CB of semiconductor to induce the reduction reaction while the photogenerated holes on the VB of semiconductor A will inject to the corresponding VB of semiconductor B to induce the oxidation reaction. For type II heterojunction, the CB and VB of semiconductor A are both higher than those of semiconductor B. Consequently, the excited electrons on the CB of semiconductor A will flow to semiconductor B. The holes generated on the VB of semiconductor B will flow to semiconductor A. The band edge alignment of Z‐scheme heterojunction is quite similar to that of type II heterojunctions. The main difference between them is the photogenerated electrons transfer pathway. In Z‐scheme heterojunctions, the electrons are generated on the CB of semiconductor B will combine with the holes generated on the VB of semiconductor A. Further, the electrons on CB of the semiconductor A will directly induce the reduction reaction while the holes on the VB of semiconductor B will lead to the oxidation reaction. Considering that the photoexcited carriers are separated from more negative and positive positions, type II and Z‐scheme systems are supposed to be more favorable for photocatalytic reaction compared to type I heterojunction.

**Figure 15:**
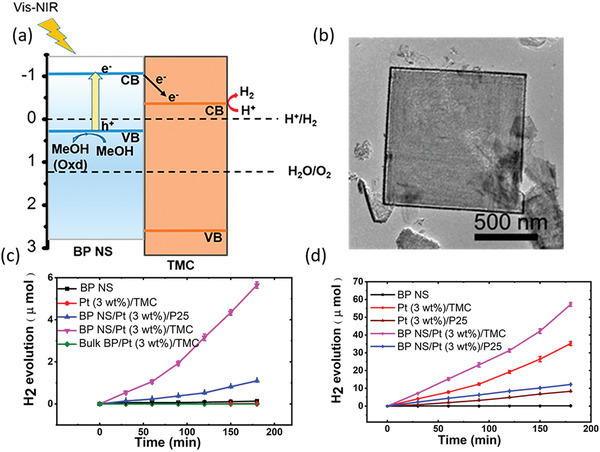
a) The charge transfer on the BP/Pt/TMC. b) TEM image of the BP/TMC. c,d) The H_2_ evolution curves of the BP/Pt/TMC photocatalyst under visible light (c) and NIR irradiation (d). a–d). Reproduced with permission.^[^ ^92^ ^]^ Copyright 2019, American Chemical Society.

Majima et al. prepared BP‐based heterojunction BP/g‐C_3_N_4_ (CN) by stirring BP nanoflakes and CN nanosheets in *N*‐methyl pyrrolidone (NMP) solution.^[^ ^32b^ ^]^ A type I heterojunction was formed at the interface between BP and CN. Electrons and holes were generated on the surface of CN under >420 nm light irradiation and then transferred to adjacent BP; thus, the recombination of the photogenerated charges in CN was inhibited. This interfacial interaction between BP and CN enhanced the trap efficiency of electrons, causing efficient H_2_ generation. The H_2_ evolution for BP/CN can reach 1.93 µmol for 3 h which is much outperformed than pristine BP. However, the separation efficient of the photogenerated carriers in type I heterojunctions is poor because both the electrons and holes transfer to the same semiconductor with a narrow bandgap. To solve this problem, a type II heterojunction was proposed. The electrons will flow from semiconductor A to semiconductor B and the holes will flow from semiconductor B to semiconductor A; therefore, the effective separation of the photogenerated charge carriers can be achieved to avoid the recombination. Various BP‐based type II heterojunctions have been widely employed for water splitting, such as BP/TiO_2,_^[^ ^92^ ^]^ BP quantum dots (BPQDs)/CN,^[^ ^93^ ^]^ and BP/CdS.^[^ ^94^ ^]^ For example, Majima et al.^[^ ^92^ ^]^ reported a type II heterojunction loaded with Pt and transition metal chalcogenide (TMC) (BP nanosheet/Pt/TiO_2_). BP/Pt/TMC heterojunction exhibits a type II charge transfer pathway as BP has a more negative CB compared with that of TMC. As **Figure** **15** shows, the electrons generated on BP nanosheets will migrate to TMC to avoid the recombination. Meanwhile, the electrons injection was induced within a very short time (a few picoseconds), thus further enhanced the separation rate for electrons and holes. The optimum H_2_ evolution rate is 1.9 and 0.41 µmol h^−1^ under visible (*λ* > 420 nm) and NIR (*λ* > 780 nm) irradiation, respectively. Besides the forming a 2D/2D heterojunction, assembling a 0D/2D type II heterojunction will render sufficient contact area and abundant active sites.^[^ ^78^, ^93^ ^]^ Liu and co‐workers^[^ ^93^ ^]^ evaluated the photocatalytic performance of a type II heterojunction by forming a 0D‐2D inorganic‐organic hybrid with BPQD and ultrathin g‐C_3_N_4_. With inherent prominent edges, the coordinately unsaturated atoms in BPQDs could hybrid with the g‐C_3_N_4_ nanosheets to form P—C covalent bonds which act as effective pathways for the carrier transportation. The photoexcited electrons on the CB of BP will migrate efficiently to the CB of g‐C_3_N_4_ nanosheets. Meanwhile, the photogenerated holes in the VB of will inject to the VB of BP. At last, an effective carrier separation was realized. The H_2_ evolution rate for BPQDs/g‐C_3_N_4_ heterojunction is 271 µmol h^−1^ g^−1^ by 5 wt%, which is 5.6 and 4.2 times larger than that of pristine g‐C_3_N_4_ nanosheets and BPQDs, respectively. Such improvement not only arises from the BPQD's intrinsic high absorption coefficient, but also results from the formation of P—C bond which can accelerate charge transfer through the P–C interface and suppress the recombination of electrons and holes.

**Figure 16:**
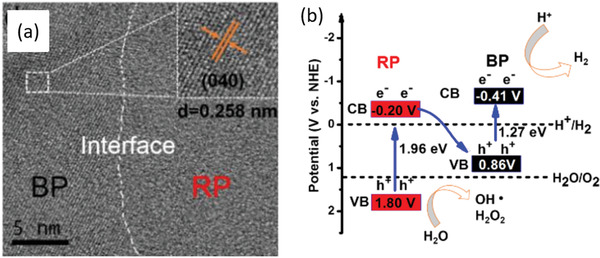
a) HRTEM image of the BP/RP heterophase junction, and b) the charge transfer on the Z‐scheme BP/RP for water splitting. a,b) Reproduced with permission.^[^ ^97^ ^]^ Copyright 2019, Wiley‐VCH.

Although type II BP‐based heterojunctions can effectively separate excited electrons and holes, the redox ability of the photogenerated charge is limited. Because after the carrier transfer, both the oxidation and reduction potential are reduced, which will weak the redox kinetics. Surprisingly, the Z‐scheme system ideally compensates the shortcomings of types I and II heterojunctions. The construct of Z‐scheme system can achieve complete water splitting reactions and efficient electrons and holes separation. Some BP‐based Z‐scheme systems, such as BP/BiVO_4_,^[^ ^47b^ ^]^ BP/Bi_2_WO_6_,^[^ ^32a^ ^]^ and BP/TiO_2_,^[^ ^95^ ^]^ have been studied. Although an effective BP/BiVO_4_ Z‐scheme system for full water splitting reactions was proved by Majima's group,^[^ ^47b^ ^]^ the H_2_ and O_2_ production rates are still low due to the poor interface contact and charge transfer efficiency between different semiconductors. Red phosphorus (RP) can form an excellent interfacial contact with BP to improve the electron transfer due to its same chemical composition with BP.^[^ ^96^ ^]^ Recently, Chen et al.^[^ ^97^ ^]^ developed a BP/RP heterojunction via wet chemistry method to achieve Z‐scheme photocatalytic water splitting. The BP/RP heterojunction was in situ grown with RP as a precursor thus a perfect interface with P‐P covalent bond at the atomic level was built between RP and BP, as shown in **Figure** **16**a. Both RP and BP were excited under visible light. The photogenerated electrons in the CB of RP can recombine with the photogenerated holes in the VB of BP due to the close band positions. Then the photogenerated electrons in the CB of BP were used for the reduction reaction, while the photogenerated holes in the VB of RP were used for the oxidation reaction. The appropriate band structures and perfect interface of BP and RP contribute to the efficient separation and transfer of the photogenerated carriers. The H_2_ production activity of BP/RP heterojunction is 0.33 mmol g^−1^ h^−1^, which is 2.5 times higher than that of the mixture of BP and RP.

As BP will easily degrade in air and water, their long‐term application in photocatalysis has been limited. Several stabilization strategies have been developed to improve its stability, including functionalization with protective atoms and hybridization with protective materials.^[^ ^98^ ^]^ Tian and co‐workers^[^ ^15^ ^]^ reported a bottom‐up chemical synthesis of BP nanosheets based on common laboratory reagents at low temperature. The obtained sample showed excellent stability due to the partial oxidation of the BP surface. The as‐prepared BP nanosheets exhibited a hydrogen production rate of 14.7 mol h^−1^ g^−1^ without any sacrificial agent, which is 24‐fold higher than the well‐known g‐C_3_N_4_ nanosheets. As O_2_ can easily decomposed on pure BP surface and oxidize BP, preventing BP form O_2_ can greatly enhance the stability of BP. Our group^[^ ^99^ ^]^ has reported a fluorinated phosphorene (FP) to prevent O_2_ from reaction with BP. An electrochemical exfoliation process by the assist of ionic liquid has been adopted for the exfoliation and fluorination of BP. Once the BP was fluorinated, the O_2_ was repelled by the highly electronegative fluorine adatoms, which will protect BP from the decomposition of O_2_. As a result, the introduction of the —F functional group greatly improved the stability of FP, and the FP exhibited excellent photothermal stability during a week of air exposure. Besides the 2D layered BP nanosheets, ultrasmall BP quantum dots (BPQDs) show a wide spectral range from the visible to the near and mid‐infrared region (NIR and MIR), which makes BPQDs a wide applications in phototherapy,^[^ ^100^ ^]^ optoelectronic devices.^[^ ^101^ ^]^ Electrochemical synthesis method can be further extended to develop highly stable fluorinated BPQDs.^[^ ^102^ ^]^

#### 4.1.2 BP for Dye Degradation

Organic pollution (such as antibiotics and dyes) have caused serious environmental pollution. Recently, a clean‐up method via photodegradation has emerged as a promising way to remove the organic dyes due to its environmentally friendly nature. Highly active reactive oxygen species (ROS), including hydroxyl (·OH), superoxide (·O_2_—), singlet oxygen (^1^O_2_), and hydrogen peroxide (H_2_O_2_), are usually used as photosensitizers owing to their strong oxidizing ability.^[^ ^103^ ^][^ ^104^ ^]^ Many materials, such as noble metals, organic molecules, as well as graphene are often employed as photocatalysts to generate ROS. However, their practical applications are usually restricted by the poor quantum yield, low photoabsorption, high cost, and so on. Recently, BP has emerged as a promising photocatalyst due to its broad light absorption high photocatalytic activity.^[^ ^8^, ^62^, ^105^ ^]^

**Figure 17:**
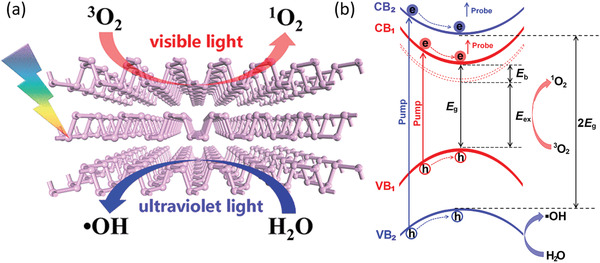
a) The illustration of optically switchable ROS generation of ^1^O_2_ and •OH. b) The electrons transfer under different light irradiation. a,b) Reproduced with permission.^[^ ^108^ ^]^ Copyright 2018, American Chemical Society.

Wang et al.^[^ ^106^ ^]^ has demonstrated for the first time that BP nanosheets’ photocatalysis efficiency for ^1^O_2_ generation under visible light irradiation. The ^1^O_2_ quantum yield of BP is ≈0.91, rendering capability in catalysis and dye degradation. Then BP's production of ·OH was further demonstrated by Yuan et al.^[^ ^107^ ^]^ In their work, BPQDs were prepared by liquid exfoliation and used as a photocatalyst for the photocatalytic degradation of RhB. The degradation efficiency of RhB is 92%, and the maximum rate constant of 0.81 h^−1^ with the BPQD photocatalyst. When BPQDs are irradiated by visible light, EHPs are generated, the electrons migrate to the surface of BPQDs and react with the absorbed O_2_ to generate O_2_^−^, and the photoexcited electrons flow to the surface‐adsorbed hydroxyl groups and water molecules, resulting the generation of ·OH radicals. Interestingly, when different light irradiation was employed, the BP exhibited a switchable photocatalytic ROS generation ability for producing ·OH and ^1^O_2_.^[^ ^108^ ^]^ As **Figure** **17**b shows, upon visible light irradiation, photoexcited holes (electrons) will transfer between the top (bottom) of VB_1_ (CB_1_) in the internal band system (red lines), leading to the generation of ^1^O_2_. When irradiated by UV‐light, the photoexcited holes (electrons) will rapidly relax to the top (bottom) of VB_2_ (CB_2_), leading to the generation of ·OH. The switchable photocatalytic ability of BP contributes to the ^1^O_2_ generation under visible light irradiation and ·OH generation under UV‐light excitation.

As the dye degradation usually involves water and oxygen, the poor stability and degradation of BP has severely restricted its long‐term applications. Combining carbon materials and BP to form a hybrid have been proved to be an effective way to improve BP's stability and photocatalytic activity.^[^ ^32^, ^76^, ^77^, ^78^ ^]^ The surface or edge of BP would be passivated by graphene, C_60_ and N‐doped graphene (NG) via covalent or noncovalent functionalization to improve the stability of BP.^[^ ^109^ ^]^Wang and his co‐workers ^[^ ^76a^ ^]^ successfully synthesized a BP/CN hybrid via ultrasonic‐assisted liquid exfoliation method in the solvent of NMP. Such 2D/2D heterostructure provides a large contact area for fast interfacial charge transfer which helps to improve the photocatalytic activity. The photoinduced electrons are apt to react with absorbed O_2_ to form ·O_2_^−^ to partially oxidize RhB. Further, the ·O_2_^−^ is further reduced by another electron and react with H+ to form H_2_O_2_. With the evolution of ·O_2_^−^ and H_2_O_2_ production, ≈98% of RhB was decomposed with 15 min of irradiation with 10% BP/CN catalyst, which is almost four times higher than that of pristine CN. Additionally, the intimate contact between BP and CN effectively passivate P atoms on the puckered surface and the CN can also act as an encapsulation reagent to protect BP from the attacks of oxygen, water and light, resulting an enhanced stability of BP in the hybrid. Another problem for BP‐based heterostructures is the weak interface between BP and semiconductors. To improve the interface quality, Yu et al.^[^ ^8a^ ^]^ prepared a BP‐RP heterostructure in which the BP grew in situ in RP to give rise to an excellent interfacial contact. Approximately 89% of RhB was degraded with 30 min of irradiation over the BP‐RP heterostructure, which is much higher than that of pure RP (57%). The photocatalytic activity of BP‐RP heterostructure is comparable to that of CdS under visible light irradiation.

### 4.2 Application of BP as an Electrocatalyst

Electrochemical methods can acquire hydrogen, oxygen as well as liquid fuels from water and CO_2_, respectively.^[^ ^81^ ^]^ However, owing to the high cost of this technology, its application in fuel generation has not been totally realized. Actually, considering that the most efficient electrocatalyst is noble metals especially Pt, quantities of efforts are devoted to find alternatives of Pt electrode. Low‐dimensional materials are regarded as the most promising candidate of catalysts owing to the key advantages of large specific surface area.^[^ ^110^ ^]^ Such advantageous property means that most of the atoms are located on the surface where catalytic reaction occurs. Thus, 2D transition metal dichalcogenides (TMDs) acquired particularly attention in electrocatalysis field.^[^ ^3^, ^111^ ^]^ Still, most of these 2D TMDs suffers from high overpotential as well as the difficulties of preparation in large scale and high quality. In addition, these transition‐metal‐based electrocatalysts exhibits high commercial cost than BP made from earth‐abundant element.

In 2016, Wang's group first reported the electrocatalytic OER activity of BP experimentally.^[^ ^2a^ ^]^ Zeng's group constructed hybrid nanosheets consisting of BP and MoS_2_ to promote electrocatalytic HER reaction in 2018.^[^ ^82^ ^]^ Their results showed that exchange current of BP/MoS_2_ was 22 times higher than MoS_2_, which was regarded as one of the most promising TMDs in electrocatalytic application. More recently, Yu's group couple BP with N‐doped graphene (NG) to construct 2D/2D heterostructure and even achieved electrocatalytic overall water splitting.^[^ ^27a^ ^]^ The two‐electrode electrolyzer with BP/NG as both anode and cathode exhibits a quite low cell voltage even smaller than the costly integrated Pt/C@RuO_2_. These meaningful results along with time evolution reflect the huge application potential of BP‐based electrocatalyst in contrast with those traditional electrocatalyst. A brief summary of BP‐based electrocatalysts for photocatalytic applications has been shown in **Table** **2**. The following sections will discuss the application of BP in electrocatalytic HER and OER field, respectively.

**Table 2:** Summary of BP‐based electrocatalysts for electrocatalytic applications (NM represents not mentioned)

Electrocatalytic applications	Samples	Electrolyte	Overpotential [mV]	Onset potential [V]	Tafel slope [mV dec^−1^]	Ref.
HER	BP NPs	0.5 m H_2_SO_4_	810		NM	^[^ ^112^ ^]^
	NH_2_‐BP	1 m KOH	290		63	^[^ ^14a^ ^]^
	BP/Ni_2_P	0.5 m H_2_SO_4_	70		81	^[^ ^113^ ^]^
	BP/Co_2_P	0.5 m H_2_SO_4_	105		62	^[^ ^42^ ^]^
	BP/Co_2_P	1 m KOH	173		72	^[^ ^42^ ^]^
	BP/NG	1 m KOH	191		76	^[^ ^109a^ ^]^
	BP/MoS_2_	0.5 m H_2_SO_4_	85		68	^[^ ^82^ ^]^
OER	BP/Ti	1 m KOH		1.48	91.52	^[^ ^2a^ ^]^
	BP/CNT	1 m KOH		1.49	72.88	^[^ ^2a^ ^]^
	BP NSs	1 m KOH		1.45	75	^[^ ^30^ ^]^
	BPQDs/CoO_x_	1 m KOH	450		58.5	^[^ ^114a^ ^]^
	BP/Co (OH)_2_	1 m KOH	276		57	^[^ ^115^ ^]^
	BP/Te	1 m KOH		1.49	NM	^[^ ^118d^ ^]^
	BP/Co	1 m KOH	310	0.21	61	^[^ ^45^ ^]^

#### 4.2.1 BP for Hydrogen Evolution Reaction (HER)

Electrochemical water splitting represents another promising approach for hydrogen generation. Although BP's potential for electrocatalytic HER has been proved by Sofer and co‐workers,^[^ ^34^ ^]^ the future for its industrial applications is still challenging. Various problems, including increasing the active sites, reducing the surface defects, and improving the hydrogen absorbing ability, still need to be solved to improve the electrocatalytic HER performance.

**Figure 18:**
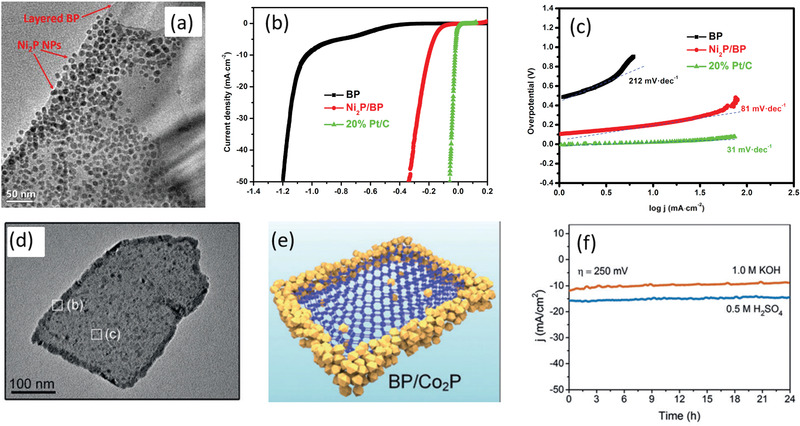
a) HRTEM of Ni_2_P/BP catalyst, b) LSV curves, c) Tafel plots for BP, Ni_2_P/BP, and 20% Pt/C catalysts. a–c) Reproduced with permission.^[^ ^113^ ^]^ Copyright 2016, Elsevier. d) TEM image and e) schematic diagram of BP/Co_2_P heterostructure, f) stability assessment of BP/Co_2_P in H_2_SO_4_ and KOH solutions. d–f) Reproduced with permission.^[^ ^42^ ^]^ Copyright 2018, Wiley‐VCH.

Improving the specific surface area of BP to expose more active sites is an efficient method to improve the electrocatalytic activity of BP. This aim can be realized by fabricating smaller size BP, such as BP nanoparticles (NPs), few‐layer BP, and BPQDs. Pumera et al.^[^ ^112^ ^]^ reported a single‐step electrochemical exfoliation to downsize bulk BP into nanoparticles. During the electrochemical exfoliation process, a potential difference was employed on the opposite ends of BP, therefore leading a fragmentation of bulk BP into nanoparticles. The resulting NPs exhibit a size distribution of 40–200 nm with an average size of 70 nm. The ultrasmall BP nanoparticles exhibit a charge transfer resistance of 1.39 kΩ compared 37.6 kΩ of BP macroparticles. The dramatic decrease of transfer resistance is in favor of carrier migration and thereby promote catalytic activity. Notably, the BP NPs show competitive performance compared with the systems reported for gold NPs. The overpotential for HER of BP NPs was −0.81 V which is lower than both that of BP macroparticles (−1.24 V) and glassy carbon (−0.97 V). Employing alien groups or molecules to interact with BP is another way to cutting the BP into small size and expose more active sites. The absorbed groups on the surface of BP can weak the interactions between BP layers and boosting the stripping effects. Chen et al.^[^ ^14a^ ^]^ reported urea‐assisted ball‐milling synthesis to prepare the few‐layer BP nanosheets (NH_2_‐BP). By introducing urea molecules, the van der Waals interactions between phosphorene layers are weakened therefore facilitate the exfoliation. In addition, the BP edges with reactive P dangling bonds can be functionalized with NH_2_ groups, which will further suppress its restacking. The thickness of the ultrathin few‐layer BP is 2.15–4.87 nm which is small enough to exhibit abundant edges and catalytic active sites. As a result, an impressive activity for HER was achieved with an overpotential of 290 mV under alkaline conditions at −10 mA cm^−2^, which is dramatically outperformed than that of bulk BP (with an overpotential of 910 mV at the same condition). Apart from cutting BP into small size, hybrid BP with catalytic active nanoparticles seems to be another feasible way to yield more active sites. Zhang et al.^[^ ^113^ ^]^ reported a hybrid of Ni_2_P nanoparticles and BP (Ni_2_P/BP) via a facile one‐step thermal decomposition method. As an excellent HER electrocatalyst, nickel phosphide has been widely used due to its small size and abundant catalytic active sites. The Ni_2_P NPs shows an ultrasmall size about 7 nm and uniformly distributed on 2D layered BP materials (shown in **Figure** **18**), which renders more catalytic active sites exposed for HER. As expected, the Ni_2_P/BP hybrid exhibits remarkable HER performance with only 185 and 227 mV overpotential to reach the current density of 10 and 20 mA cm^−2^, respectively. The catalytic activity for HER is superior than that of pure Ni_2_P NPs (*η*_10_ = 213 mV), and many other non‐noble‐metal HER electrocatalysts, such as MoS_2_ NSs (*η*_10_ = 195 mV) and FeP NSs (*η*_10_ = 240 mV). Considering that many defects are inevitably produced on the surfaces and edges during the synthesis process, the stability, conductivity and electrochemical activity of BP is severely undermined. To cope with the defects, Yu's group^[^ ^42^ ^]^ utilized the reactive edge defects on BP nanosheets as the initial sites to grow Co_2_P nanocrystals via a simple solvothermal method. The defects on BP result in unsaturated P atoms with smaller coordination number as well as higher reducing activity. In addition, the defects exhibit a lower occupation energy of Co (−7.65 eV) than the surface adsorption energy (−4.41 eV). As a result, Co ions can be easily reduced to Co atoms and bond with adjacent P atoms to grow Co_2_P nanocrystals on the edge defects (Figure 18d,e). At last, the defects on the edges of BP were occupied to passivate the degradation. Meanwhile, an in‐plane BP/Co_2_P heterostructure was formed to improve the electrocatalytic properties due to the Co_2_P serve as active sites and suppress the stacking of the nanosheets to expose more active sites in in‐plane direction. BP/Co_2_P shows an overpotential of 105 mV to reach 100 mA cm^−2^ in 0.5 m H_2_SO_4_ and an overpotential of 173 mV for 100 mA cm^−2^ in 1.0 m KOH, which is much lower than that of BP with 389 and 336 mV under the same conditions. Moreover, BP/Co_2_P also exhibits superior stability by remaining constant in 0.5 m H_2_SO_4_ and 81% in 1.0 m KOH after electrolysis for 24 h.

BP has a large Gibbs free energy (∆*G*_H*_ = 0.67 eV), which correspond to very weak H adsorption and limits its electrocatalytic ability for HER. Both carbon and transition metals materials have moderate Gibbs free energy closed to zero which could facilitate the adsorption and desorption of hydrogen. In this regard, doping BP with these materials is a promising strategy to balance the hydrogen adsorption and desorption ability. Dai et al.^[^ ^109a^ ^]^ reported an N‐doped graphene (NG) 2D/2D heterostructure with few‐layered exfoliated BP (EBP) nanosheets (EBP@NG heterostructure). As NG exhibits a higher Fermi level than EBP nanosheets, photogenerated electrons can transfer from NG to EBP nanosheets, enabling more electrons on EBP where the proton (H+) obtains an electron to form a H adatom that binds to the surface of EBP. Therefore, the Δ*G*_H*_ will decrease via electrons accumulation. The ∆*G*_H*_ of pristine BP shifted closer to zero (∆*G*_H*_ = 0.1 eV) when coupled with NG, which is in favor of HER activity due to balanced hydrogen adsorption and desorption ability. EBP@NG exhibits a dramatically improved HER activity with a low overpotential of only 191 mV to achieve current density of 10 mA cm^−2^, which is much lower than that of pure EBP (370 mV) or NG (445 mV) under the same conditions. Molybdenum disulfide (MoS_2_) has an outstanding HER catalytic activity due to its moderate free energy of H adsorption. A MoS_2_ doped nanosheets with BP was synthesized by Zeng's group^[^ ^82^ ^]^ to enhance the HER activity. BP has a higher Fermi level than MoS_2_, and the electrons on BP nanosheets will inject to MoS_2_ to reduce the Δ*G*_H*_ of MoS_2_. As a result, the intrinsic exchange current density *j*_0_ increase to 0.66 mA cm^−2^, which was 22 times higher than that of pristine MoS_2_. Therefore, the original active sites in MoS_2_ are activated enormously. As expected, the MoS_2_–BP nanosheets exhibited high catalytic performance for HER with an overpotential as low as 85 mV at 10 mA cm^−2^, which is much lower than the pure BP (161 mV) and MoS_2_/C (117 mV). Moreover, the electrocatalytic stability of MoS_2_–BP nanosheets was also improved through the construction of MoS_2_‐based hybrid, indicating its potential for long term application in HER field.

#### 4.2.2 BP for Oxygen Evolution Reaction (OER)

The kinetics of OER is sluggish as the breaking of O—H bonds require certain energies to produce oxygen. Compared to two electrons transfer pathway in HER to produce H_2_, four electrons transfer pathway is desired in OER to produce O_2_. Thus, a much higher overpotential is demanded to overcome the kinetic barrier for water oxidation to generate O_2_. The threshold is to develop a highly efficient catalyst with high activity and excellent stability. Currently, precious‐metal‐based materials, such as IrO_2_ and RuO_2_, are widely used as catalysts for OER. However, these materials are of high cost and scarce for large‐scale production. Recently, tremendous efforts have been made on the search for metal‐free catalysts with low cost and highly catalytic active merits. As a promising candidate for highly efficient metal‐free catalyst, BP has attracted much attention due to its high carrier mobility (200 cm^2^ V^−1^ s^−1^), efficient catalytic activities and low cost. Tremendous applications for BP have been applied in photocatalysis, dye degradation and electrocatalytic HER as mentioned above. Nevertheless, relatively little has been reported on BP‐based catalysts for OER applications. As Wang et al.^[^ ^2a^ ^]^ demonstrated for the first time the electrocatalytic OER activity of BP nanosheets, the promise for its OER applications have just been implemented. In their study, BP was grown on electrically conductive Ti substrate for electrocatalytic evaluation. The BP‐Ti exhibits an intrinsic activity with a Tafel slope of 91.52 mV dec^−1^ and a relatively small charge transfer resistance (*R*_ct_ = 263.4 Ω). However, due to its 2D structure, the BP‐Ti thin‐film hybrid leads to limited exposed surface sites, poor conductivity and exposed lone pairs electrons which hinder its durability for long‐term OER catalysis. For this reason, recent strategies to enhance BP's durability and efficiency for OER are based on increasing active sites, building highly conductive interface for electron transfer and occupying the lone pairs to acquire long‐term stability.

**Figure 19:**
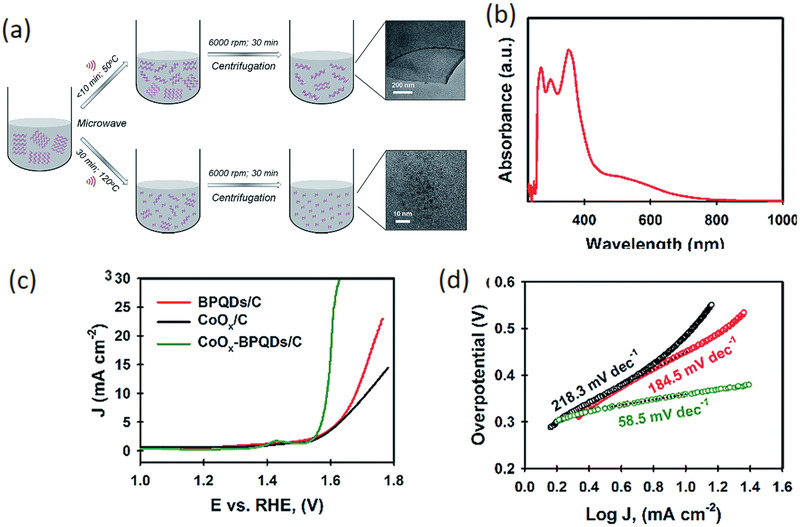
a) Schematic illustration of the MW‐assisted preparation for BP nanosheets and BPQDs. b) UV–vis spectrum of BPQDs, c) LSV curves, and d) Tafel plots of the BPQDs/C (red), CoO_*x*_/C (black) and CoO_*x*_–BPQDs/C (green) electrocatalysts for OER measured in 1.0 m KOH electrolyte. a–d) Reproduced with permission.^[^ ^114a^ ^]^ Copyright 2019, The Royal Society of Chemistry.

Reducing the thickness of BP nanosheets is widely used to generate more active sites for OER. Zhang’ group^[^ ^30^ ^]^ reported a liquid exfoliation route to prepare 2D few‐layer BP nanosheets and the ultrathin BP NSs with different sizes can be obtained by various centrifugation speeds. The average thickness of BP nanosheets are 70, 54, 22, and 6 nm under the centrifugation speed of 0–500, 500–1000, 1000–1500, and above 1500 rpm, respectively. The current density at 1.0–1.8 V is increasing with the BP NSs’ centrifugation speed. When the centrifugation speed is lower than 1000 rpm, the current density is even lower than the bulk BP (6.1 mA cm^−2^). By increasing the centrifugation speed to 1500 rpm, the electrocatalytic activity for OER is dramatically increased with a current density of 19.6 mA cm^−2^. Moreover, a lower Tafel slope of 75 mV dec^−1^ was also observed for thickness distributions of “above 1500 rpm” compared to the pristine BP and nanosheets obtained under “1000–1500 rpm” (88 mV dec^−1^). Smaller size contributes to more active sites in the edge plane of BP nanosheets. Therefore, reducing the thickness benefits a better OER electrocatalytic activity of BP nanosheets. With an even smaller size than BP NSs, the BPQDs exhibit great potential in electrocatalysis because of their high conductivity and abundant electrocatalytic active sites.^[^ ^114^ ^]^ However, an efficient and facile synthetic approach to produce high quality BPQDs for OER is still challenging. Batmunkh et al.^[^ ^114a^ ^]^ proposed a novel approach to synthesize highly crystalline BPQDs via a novel microwave‐assisted liquid‐phase exfoliation. The van der Waals interaction between the BP layers has been weakened through microwave in *N*‐methyl‐2‐pyrrolidone (NMP), resulting in effective layer exfoliation. Further, by increasing the exfoliation time (to 30 min) and temperature (to 120 °C), ultrasmall BPQDs were obtained. The synthesis process is illustrated in **Figure** **19**. The average lateral size and thickness of the as‐prepared BPQDs is 2.95 and 3.59 nm, respectively. The high quality BPQDs provide an impressive catalytic activity due to their strong visible light absorption and abundant electrocatalytic active sites for OER. As shown in Figure 19c,d, the overpotential is only 450 mV to achieve 10 mA cm^−2^ without any cocatalyst, which is much outperform than BP nanosheets and comparable to that of commercial CoO*_x_* electrocatalyst (480 mV). Moreover, the OER activity can be further improved by incorporating with CoO*_x_* which possessing inherently excellent electrocatalytic activity. The BP‐CoO*_x_* shows a dramatically enhanced electrocatalytic performance with a low overpotential (360 mV). The Tafel slope (mV dec^−1^) is significantly lower than that of the individual BPQDs and CoO*_x_* due to the outstanding synergistic effect between BPQDs and CoO*_x_*.

**Figure 20:**
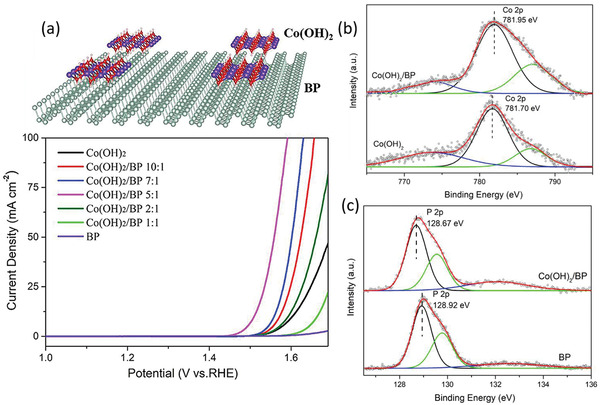
a) Scheme of the construction and polarization curves Co(OH)_2_/BP with different mass ratio. b,c) The high‐resolution XPS spectra for Co 2p (b) and P 2p (c). a–c). Reproduced with permission.^[^ ^115^ ^]^ Copyright 2018, Elsevier.

With a large surface area and high conductivity, carbon nanotube (CNT) was utilized as a substrate helping to provide more active sites and improve BP's conductivity. The BP‐CNT hybrid was prepared by a thermal‐vaporization transformation (TVT) method.^[^ ^2a^ ^]^ The vaporized P species can easily distribute in carbon nanotube network to form a 3D porous structure due to BP's strong adsorption of carbon materials. The BP‐CNT exhibits an excellent catalytic performance with a lower Tafel slope (72.88 mV dec^−1^) and charge transfer resistance (*R*_ct_ = 191.4 Ω). And the required potential is 1.6 V at 10 mA cm^−2^ is, which is comparable to that of commercial IrO_2_ (1.57 V) and RuO_2_ (1.59 V) electrocatalysts. The observed superior OER performance of BP‐CNT can be attributed to the porous structure with more active sites and highly conductive CNT. However, instead of forming a chemical binding of B–C, P atoms are physical adsorbed within CNT which provides a poor efficient electron transfer pathway for electrocatalytic OER. To construct a highly conductive and stable interface, Li and co‐workers^[^ ^115^ ^]^ have deposited Co(OH)_2_ nanosheets on BP nanosheets via a stirring mixing method, as shown in **Figure** **20**a. According to the XPS results, a covalent bond Co–P was formed and the optimized inter layer spacing is only 2.2 Å, which is much smaller than BP/graphene (3.49 Å), BP/hexagonal boron nitride (3.46 Å) and BP/MoS_2_ (3.30 Å). The strong inter layer binding energy and small inter layer distance is in favor of fast and efficient charge carriers transfer. As Co (OH)_2_ possesses a higher Fermi level than BP, abundant electrons will migrate from Co(OH)_2_ to BP, leading to superior catalytic activity for OER. The lowest overpotential for the Co(OH)2/BP is 276 mV at 10 mA cm^−2^ and a small Tafel slope of 57 mV dec^−1^_,_ which is greatly outperformed than that of Co(OH)_2_ (370 mV, 88 mV dec^−1^).

Additionally, various kinds of vacant defects are unavoidable in BP layers during fabrication, leaving tremendous exposed lone pair electrons which is the major reason for degradation. Many reports have focused on preparing high quality BP NSs to coping with the defects. ^[^ ^116^ ^]^ Huang et al.^[^ ^116a^ ^]^ proposed an electrochemical cations intercalation method to prepare phosphorene, in which tetrabutylammonium cations were utilized as electrolyte. The applied potential boosts the structural expansion and cationic insertions, which facilitated the preparation process (less than 1 h) and reduced the extensive defects resulting from the ultralong sonication process in traditional liquid exfoliation. Moreover, the cationic insertions without oxidizing conditions would avoid the decoration with oxygen functional groups to obtain a defect free crystalline structure. CVD method represents another approach to producing high quality 2D materials such as graphene, h‐Bn, and TMDs.^[^ ^117^ ^]^ Although an in situ CVD‐type approach for growing BP nanosheets from red phosphorus has been demonstrated,^[^ ^116c^ ^]^ the successful CVD growth for 2D BP still remains unexplored. Recently, some researchers have proposed strategies to take advantages of the defects as active sites for elements doping^[^ ^118^ ^]^ and structural construction to realize long‐term durability.^[^ ^45^, ^115^ ^]^ A series of defects, including Stone–Wales, single vacancy defects and zigzag nanoribbon, are usually obtained on the BP nanosheets. The formation energies of tellurium (Te) dopant for Stone–Wales, single vacancy defects and zigzag nanoribbon are greatly reduced compared to pristine phosphorene, indicating the Te atoms are much more likely to introduced into the lattice when various defects exist in phosphorene.^[^ ^118b^ ^]^ Moreover, due to the dangling bonds of edge P atoms on the zigzag nanoribbon edge, the Te atoms are more likely to bond with P atoms. As a result, the Te atoms can be readily stabilized by taking advantages of the intrinsic defects on BP NSs. Benefiting from the moderate adsorption energy of O*, Te atoms can further serve as active sites to reduce the strong adsorption strength of O* on BP NSs, which are crucial for improving its OER catalytic activity.^[^ ^118c^ ^]^ The enhanced electrocatalytic OER activity of BP NSs was proved with an lower onset potential of 1.49 V compared to the 1.63 V of undoped BP NSs.^[^ ^118d^ ^]^ To deal with the exposed lone pair electrons, a Co/BP NSs hybrid electrocatalyst was constructed.^[^ ^45^ ^]^ Owing to higher Fermi level of BP than that of Co NPs, the active lone pair electrons will transport from BP to Co, protecting BP NSs from degradation and simultaneously retaining their high hole mobility. Co/BP NSs shows an enhanced OER activity with a low overpotential (310 mV at 10 mA cm^−2^), and a small Tafel slope (61 mV dec^−1^). According to the cyclic voltammetry (CV) test and constant potential test, the Co/BP NSs exhibit excellent stability in 1000 cycles (with a mere 16 mV shift at 50 mA cm^−2^) and maintain an unchanged electrochemical activity in 55 h.

## 5 Applications of BP‐Analog Materials in Catalysis

In recently years, 2D materials such as graphene and transition metal dichalcogenides (TMDs) have become the focus of research due to their unique structural and optical/electro properties.^[^ ^119^ ^]^ Although much progress on 2D materials has been made in batteries, catalysts, optoelectronics devices, and other applications.^[^ ^120^ ^]^ the progress on those materials were still limited due to the zero bandgap of graphene and low current mobility of TMDs. Furthermore, the large bandgap of TMDs leads to a poor photocatalytic efficiency in the vis–NIR region.^[^ ^121^ ^]^ Surprisingly, the emergence of and BP (with a tunable bandgap of 0.3–1.5 eV) bridges in the gap between graphene and TMDs, which renders BP's strong absorption in vis–NIR region. However, due to its degradation nature and low separation efficiency for photo/electrogenerated charges, its applications in photo/electrocatalysis has been severely limited. Recently, BP‐analog materials, including 2D binary IV–VI compound,^[^ ^122^ ^]^ layered metal phosphate materials,^[^ ^123^ ^]^ 2D monoelemental BP‐analog materials,^[^ ^124^ ^]^ have attracted much attention because of its analog structures and proprieties with BP but much improved stability, high mobility and diversity than BP. In this part, we will introduce the structure, properties, and applications of BP‐analog materials in the regard of catalysis.

### 5.1 2D Binary IV–VI Compound

#### 5.1.1 Structures and Properties

**Figure 21:**
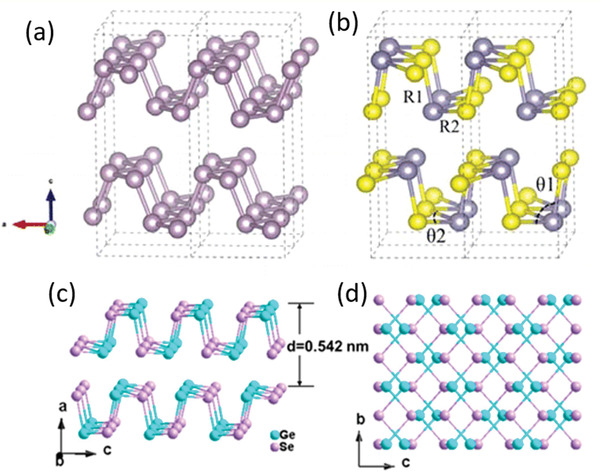
a,b) Crystal structures of BP (a) and SnS (b). a,b). Reproduced with permission.^[^ ^125a^ ^]^ Copyright 2016, American Chemical Society. c) GeSe (side view) and d) GeSe (top view). c,d) Reproduced with permission.^[^ ^125b^ ^]^ Copyright 2017, American Chemical Society.

2D binary IV–VI compound, including tin sulfide (SnS), germanium sulfide (GeS), germanium selenide (GeSe), and tin selenide (SnSe), features a wrinkled and layered structure which is analog to BP, as shown in **Figure** **21**.^[^ ^125^ ^]^ The in‐plane atoms combine with each other by strong covalent bond, with 100% exposed surface atoms that could serve as active sites for catalyst. The out‐of‐plane layers tacked together by weak van der Waals force, makes the 2D binary IV–VI compound easily exfoliated to few layers nanosheets. Similar to BP, the wrinkled structure leads to strong in‐plane anisotropy in optoelectronic and mechanical properties. SnS is the most widely studied BP analog 2D binary IV–VI compound due to its unique structure and properties. SnS has an indirect bandgap that can be tuned from 1.07 to 1.25 eV and a direct bandgap that can be tuned from 1.30 to 1.39 eV.^[^ ^122^ ^]^ Correspondingly, it has a wide absorption ability from ultraviolet to near infrared regions, which is comparable to that of BP. Moreover, SnS also exhibits a high absorption coefficient (5 × 10^4^ cm^−1^). Its intrinsic p‐type conductivity renders an effective electron transfer for hydrogen reduction and the conductivity type can be further tuned by Sn/S ratio. The ultrahigh carrier mobility (10 000–38 000 cm^2^ V^−1^ s^−1^) of SnS is beneficial to the fast separation for photogenerated carriers.^[^ ^125a^ ^]^ Last but not the least, SnS has a better ambient stability than BP and thereby could be used for long term catalytic applications.^[^ ^126^ ^]^ All these properties indicates a promising future of SnS for photo/electrocatalysis.

#### 5.1.2 Applications in Catalysis

**Figure 22:**
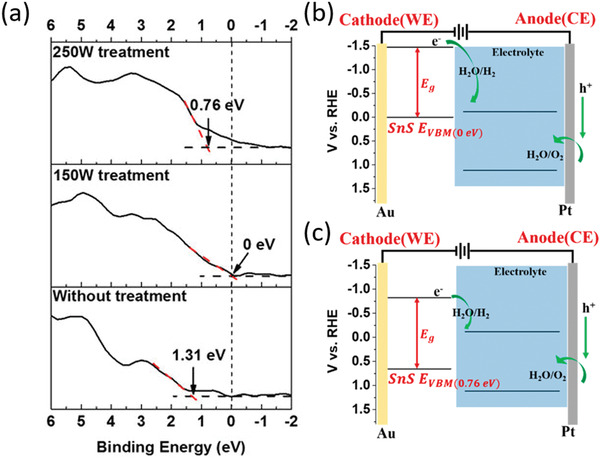
a) Valence band maxima (VBM) fitting before and after AAPT. b,c) Schematic energy band diagrams of SnS with 150 W (b) and 250 W (c) AAPT. a–c) Reproduced under the terms of the CC‐BY Creative Commons Attribution 4.0 International License (http://creativecommons.org/icenses/by/4.0).^[^ ^128a^ ^]^ Copyright 2018, The Authors, published by MDPI.

With outstanding catalytic activity and electrical conductivity, SnS has been widely utilized as photocatalysts,^[^ ^127^ ^]^ electrocatalysts,^[^ ^128^ ^]^ and electrodes for Li‐batteries.^[^ ^129^ ^]^ However, the pristine SnS shows weak catalytic activity due to its low effective carrier density. To improve its catalysis activity, creating more active sites on the surface can facilitate charge transfer. Many strategies have been adopted, such as surface functional modification,^[^ ^128a^ ^]^ and hybridizing with various materials.^[^ ^128c^ ^]^ Huang et al.^[^ ^128a^ ^]^ applied an atmospheric air plasma treatment (AAPT) method to enhance the HER performance of SnS thin films by creating more active sites on the surface of SnS. The evaporation of S atoms in SnS generates numerous surface S‐vacancies under plasma treatment. With a moderate plasmas power of 150 W, the S‐vacancies yield active edge sites which can boost the HER activity. When the power was further increased to 250 W, the surface oxidation may emerge. The VBM of SnS can also be tuned under AAPT along with different plasma power. The VBM of pristine SnS shifted from 1.31 to 0 eV and 0.76 eV under 150 and 250 W AAPT, respectively, rendering a p‐type pinning with the CBM of SnS shifting to a higher position than hydrogen reduction level (shown in **Figure** **22**). The current density under the potential of −0.325 V increased from −0.224 to 10 mA cm^−2^ after 150 W AAPT, showing a dramatic enhancement for electrocatalytic performance. However, the current density decreased to −0.394 mA cm^−2^ for 250 W AAPT under the same condition. The electrocatalytic activity enhancement for 150 W AAPT can be ascribed to the presence of the Sn active edge sites and formation of p‐type pinning, while the deterioration performance for 250 W AAPT may resulted from the overoxidation on SnS thin films. Besides the surface modification, hybriding SnS with conducting carbon materials has been proven to be another effective way to improving their HER performance. Lee et al.^[^ ^128c^ ^]^ has grown SnS nanoparticles on N‐reduced graphene (N‐rGr) sheets to form a strong SnS/N‐rGr chemical/electronic coupling. According to the turnover frequency (TOF) examination, the small size SnS on graphene yields abundant exposed edges and all the surface sulfur sites participated in the HER catalysis, rendering a high content of active sites. As expected, the SnS/N‐rGr hybrid shows high HER performance with small onset overpotential of −0.125 V and a Tafel slope of 38 mV dec^−1^, suggesting SnS's capability for electrocatalytic water splitting.

**Figure 23:**
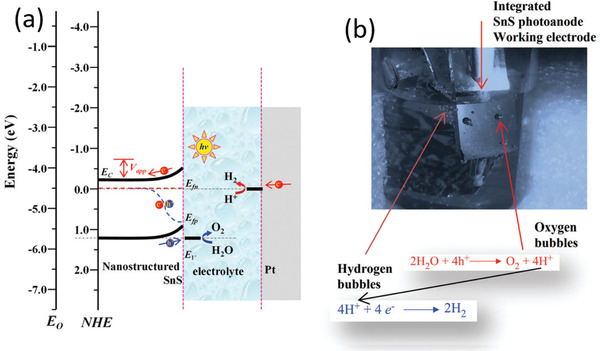
a) The bands position of SnS relative to water splitting reactions. b) The water splitting phenomena in constructed PEC cell. a,b) Reproduced with permission.^[^ ^127^ ^]^ Copyright 2016, The Royal Society of Chemistry.

SnS can also be used as a photocatalyst due to its strong optical absorption ability and high mobility (2.37 × 10^4^ and 7.35 × 10^4^ cm^2^ V^−1^ s^−1^, respectively). Patel et al.^[^ ^127^ ^]^ synthesized SnS thin films by chemical spray pyrolysis and studied its potential for photocatalyst. The as‐prepared SnS has a direct bandgap of 1.42 eV with a high solar absorption coefficient up to 2.7 × 10^5^ cm^−1^, indicating its excellent absorption ability in visible range. For measuring the photoactivity of SnS, a semiconductor‐based photoelectrochemical (PEC) cell consists of a SnS photoanode was developed. The as prepared PEC cell exhibits a photocurrent density up to 7 mA cm^−2^ at an applied potential of 0.8 V versus Ag/AgCl. The SnS exhibits a perfect intrinsic bandgap structure for full water splitting as shown in **Figure** **23**a. The respective conduction band minimum (CBM) of SnS lies at a more negative potential than the reduction potential of hydrogen evolution reaction, while the valence band maximum (VBM) of SnS lies at a more positive potential than the oxidation potential of oxygen evolution reaction. Thus, a full water splitting reaction can be obtained. The evolved oxygen and hydrogen gases on the electrode was observed and the reaction mechanism is shown in Figure 23b. This performance is much outperformed than the BP nanosheets, which can only drive half of the water splitting reaction. Besides SnS, other BP analog 2D binary IV–VI compounds, such as GeS, SnSe, and GeSn can also be applied in the catalysis applications.^[^ ^125b^ ^]^ GeS shows a high HER and ORR activities while a low OER activity. ^[^ ^130^ ^]^ However, few studies have been conducted to explore the potential of new BP analog 2D binary IV–VI compounds for catalytic application because of their current poor catalytic activity. As the electrochemical performance of the compounds can be enhanced by various structural engineering, there is a big room for exploring new BP analog 2D binary IV–VI compounds for related applications.

### 5.2 Layered Metal Phosphite Materials

#### 5.2.1 Structure and Properties

**Figure 24:**
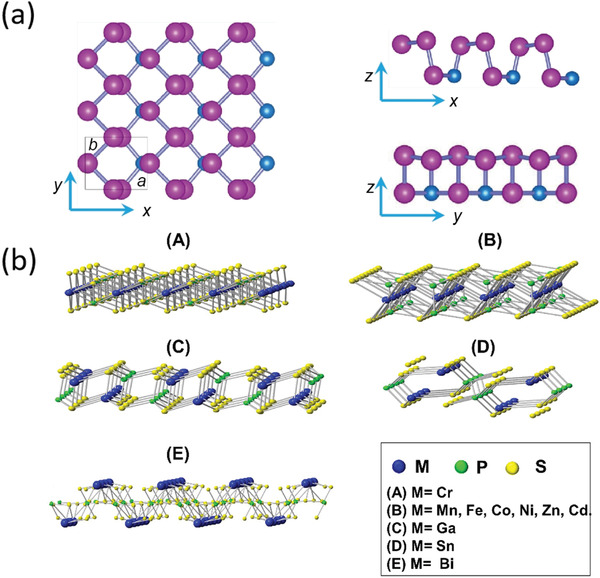
a) Top view and side view of the crystal structure of MPn Sb_0.75_P_0.25_. Reproduced with permission.^[^ ^131b^ ^]^ Copyright 2016, Science China Press and Springer‐Verlag Berlin Heidelberg. b) Side view of the crystal structures MPX*_n_*. Reproduced with permission.^[^ ^123b^ ^]^ Copyright 2017, American Chemical Society.

The layered metal phosphite materials include binary compounds MP*_n_* ^[^ ^131^ ^]^ and thiophosphite materials (MPX*_n_*).^[^ ^123b^ ^]^ For MP*_n_*, M represents element in groups IIIA, IVA, and VA. Different structures can be formed when incorporated with various M elements. For example, the P atoms in phosphorene can be substituted by boron atoms, antimonide atoms, or cobalt atoms to form BP,^[^ ^131a^ ^]^ As*_x_*P_1−_ *_x_* ^[^ ^131b^ ^]^ or CoP.^[^ ^132^ ^]^ Similar to black phosphorous, the MP*_n_* remains the hexagonal and layered geometrical structure, as shown in **Figure** **24**a.^[^ ^131b^ ^]^ With similar configurations with BP, 2D MP*_n_* are supposed to possess analog properties with BP, such as anisotropic properties, tunable bandgap, high carrier mobility, large capacity and high absorbance. Moreover, the MP*_n_* shows an excellent stability which may be suitable for long life photocatalytic or electrocatalytic applications. For transition metal phosphorus trichalcogenides MPX*_n_*, M represents the elements in group IVB, VB, and VIB, while X denotes S or Se. The family of MPX*_n_* possesses various chemical diversity and structural complexity as compared to the conventional binary compounds MP*_n_*. The various M element in MPX*_n_* materials results in multiple crystal structures. For example, when incorporated with transitional metals (Mn, Fe, Co, Ni, Zn, and Cd), MPX*_n_* exhibits a monoclinic structure (space group C2) (Figure 24b).^[^ ^123b^ ^]^ Every M atom coordinated with six X atoms, and the P atoms are tetrahedral coordinated with three X atoms to form a [P_2_X_6_]^4−^ unit, each unit is located in the center of the neighboring six M atoms, which are formed a hexagonal structure. When p‐metal (Ga, Sn, Bi) is hybridized, various crystal structures have emerged, such as monoclinic structure (space group P2_1/c_) and orthorhombic structure (space group *Ibca*). The multiple crystal structures emanate from different MPX*_n_* materials lead to versatile catalytic properties. The transitional metals MPX*_n_* materials, possess a strong preferential orientation in (001) which is believed to be highly activity for HER. And the MPX*_n_* materials with induced cobalt elements result in excellent OER activity.^[^ ^123b^ ^]^ Similar to other 2D materials, MPX*_n_* shows layered structure with weak van der Waals interactions, which indicates the materials are easy to be exfoliated into mono or few layers. The previous reports indicated that the bandgaps of these MPX*_n_* range from 1.3 to 3.5 eV, suggesting a broad wave length light absorption for photocatalysts.^[^ ^123a^ ^]^

#### 5.2.2 Applications in Catalysis

**Figure 25:**
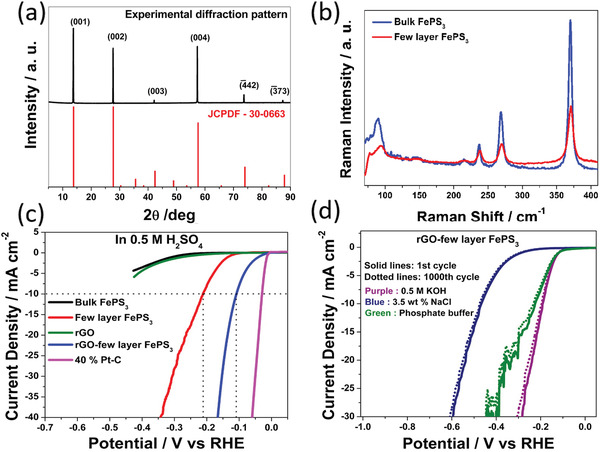
a) XRD pattern of bulk FePS_3_ with corresponding JCPDF pattern (30‐0663); b) Raman spectra of bulk and few layer FePS_3_; c) the HER activity for various catalysts in 0.5 m H_2_SO_4_; and d) electrochemical durability of rGO‐few layer FePS_3_ in alkaline, acidic, and neutral solutions. a–d) Reproduced with permission.^[^ ^123c^ ^]^ Copyright 2016, American Chemical Society.

For transitional metals MPX*_n_* materials, both S and P elements are expected to be favorable for H adsorption and desorption.^[^ ^123c^ ^]^ Presence of S element can tune the electroactivity of MoSe^[^ ^133^ ^]^ while P doped CoS_2_ or MoS_2_ are excellent catalysts for HER.^[^ ^134^ ^]^ Moreover, increasing the atomic content of P can also increase the HER activity of in transition metal phosphides, such as Ni_*x*_P_*y*,_^[^ ^135^ ^]^ Mo_*x*_P_*y*,_^[^ ^136^ ^]^ and Co_*x*_P_*y*._^[^ ^132^ ^]^ Further, the thiophosphite material MPS*_x_* (CoPS, PdPS, FePS_3_) contains both S and P element which are postulated to be highly active for HER.^[^ ^137^ ^]^ As Fe is a low cost metal with abundant earth storage, hybrid Fe metal with S and P elements can realize the large scale production of HER catalysts. In this regard, the catalytic capability of FePS_3_ for HER has been explored.^[^ ^123c^ ^]^ According to the XRD and Raman results in **Figure** **25**, the FePS_3_ is highly crystalline, and a P—S bond from [P_2_S_6_]^4−^ unit was formed. S is abundant on the surface and act as adsorption sites for H atoms. Moreover, FePS_3_ exhibits a layer stacked structure by weak van der Waals force, indicating its capability to form 2D materials to expose more active sites. Many other catalysts, such as Ni_2_P, MoP, and Mo_2_C,^[^ ^138^ ^]^ show good activity in alkaline medium but degrade quickly in acidic condition which hinders their applications. Interestingly, FePS_3_ exhibits great HER activity and catalytic stability in a wide PH range from alkaline to acidic conditions. Its structure and composition remain unchanged after 1000 cycles.

**Figure 26:**
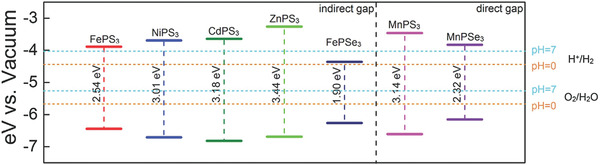
The location of VBM and CBM of various MPS3 and MPSe3 monolayers. Reproduced under the terms of the CC‐BY Creative Commons Attribution 4.0 International license (https://creativecommons.org/licenses/by/4.0).^[^ ^141^ ^]^ Copyright 2016, The Authors, published by Wiley‐VCH.

Preparing ultrathin and high quality 2D MPX*_n_* materials is another challenge of enhancing the catalytic activity. The method employed so far such as chemical vapor transport (CVT) process, require high temperature (>600 °C) and long synthesis time (up to couple of weeks).^[^ ^139^ ^]^ Serendipitously, chemical vapor deposition (CVD) emerged as a facile way to swiftly grow few‐atomic layered MPX*_n_* materials in relatively low temperature. He and co‐workers^[^ ^140^ ^]^ have adopted CVD process to grow ultrathin nickel phosphorus trisulfide (NiPS_3_). The as‐prepared NiPS_3_ hexagonal nanosheet shows few atomic layers (≤3.5 nm) and lateral size of >15 µm. The NiPS_3_ nanosheet also shows a clean surface with high crystalline quality, which is favorable to electron transfer. Moreover, its perfect band edge position ensures its feasibility as a photocatalyst in visible light region. The bandgap of NiPS_3_ is 1.96 eV and the CB level of NiPS_3_ (−3.61 eV) is situated above the H^+^/H_2_ potential, rendering its ability for solar light absorption and H_2_ generation. The constant H_2_ evolution rate is calculated as 6.46 µmol g^−1^ h^−1^. However, most of the MPX*_n_* materials exhibit an indirect bandgap which hinders their optical absorption ability. MnPS_3_ and MnPSe_3_ are found to possess intrinsic direct bandgap among the MPX*_n_* family, as shown in **Figure** **26**.^[^ ^141^ ^]^ Furthermore, their conductive band level is higher than H^+^/H_2_ potential in both acidic and neutral condition, indicating their broad applicability for photocatalytic water‐splitting. The electron mobility of MnPSe_3_ is up to 625.9 cm^2^ V^−1^ S^−1^ which is beneficial to the electron migration. However, the hole mobility on the surface is only 34.7 cm^2^ V^−1^ S^−1^. The strong anisotropic mobility will contribute to an effective separation for electrons and holes. Similar to other 2D photocatalysts, ultrathin MnPS_3_ and MnPSe_3_ nanosheets yield high specific surface area and short distance for carrier migration. But the synthesis of ultrathin MnPSe_3_ and MnPS_3_ with high crystal quality remains a great challenge.^[^ ^123d^ ^]^

### 5.3 2D BP Analog Monoelemental Materials

#### 5.3.1 Structure and Properties

**Figure 27:**
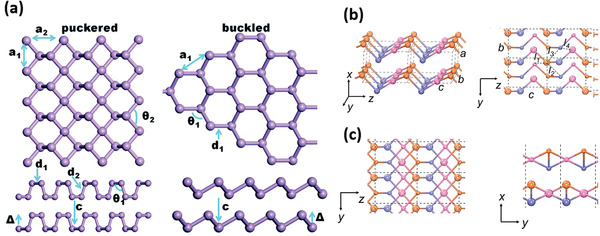
a) The atomic structures of orthorhombic and rhombohedral of pnictogens. Reproduced with permission.^[^ ^143^ ^]^ Copyright 2019, The Royal Society of Chemistry. b,c) Top and side‐views of the crystal structures of *α*‐Te (b) and *β*‐Te (c). b,c). Reproduced with permission.^[^ ^145^ ^]^ Copyright 2018, Elsevier.

**Figure 28:**
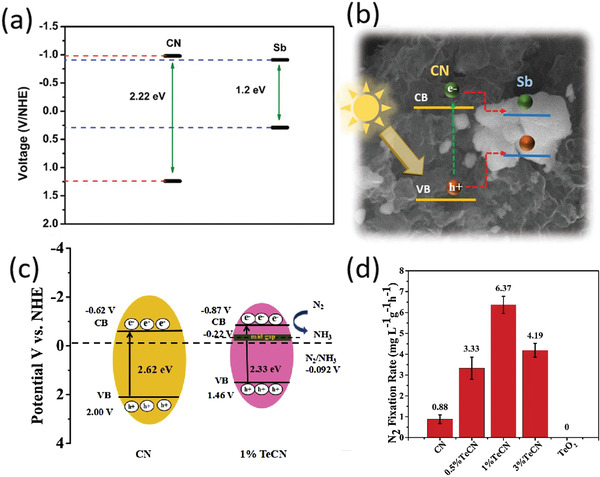
a) Band structure of the CNSb*_x_* heterostructure, and b) illustration for the photocatalytic RhB degradation on CNSb*_x_* heterostructures. a,b) Reproduced with permission.^[^ ^150^ ^]^ Copyright 2018, Wiley‐VCH. c) The photocatalytic mechanism of TeCN. d) photocatalytic nitrogen fixation rates for CN, TeO_2_, and TeCN. c,d) Reproduced with permission.^[^ ^152^ ^]^ Copyright 2018, Elsevier.

The 2D BP analog monoelemental materials include two kinds of materials which are called pnictogens and chalcogens. The pnictogens refer to the fifth main group elements (P, As, Sb, Bi) in which the heavy pnictogens (As, Sb, and Bi) share the same group with phosphorus. There are two types of crystal structure for heavy pnictogens: an orthorhombic (also termed as *α* phase) and a rhombohedra (also termed as *β* phase) structure.^[^ ^142^ ^]^ As shown in **Figure** **27**a,^[^ ^143^ ^]^ the *α* phase, features a layered structure with puckered six‐member rings which is similar to the black phosphorous. The *β* phase features a buckled layer structure which is similar to the blue phosphorous. The difference between puckered and buckled structures result in different atomic orbitals interaction, which significantly influence not only the in‐plane anisotropy but also the interlayer interactions. For *α* phase pnictogens, each layer is stacked by van der Waals force due to large out‐of‐plane interatomic distances while for *β* phase pnictogens, the individual layer is held together by much stronger interactions. The chalcogens refer to the sixth main group of elements (Se, Te). The chalcogens possess honeycomb structure stacked by vdWs forces, which makes them easy to be exfoliated into 2D materials. Similar to BP, the chalcogens feature tunable bandgap, strong anisotropy and carrier mobility.^[^ ^144^ ^]^ The bulk Te exhibits a parallel‐aligned triangle chains with three Te atoms in each unit, which is termed as *α* phase (shown in Figure 27b).^[^ ^145^ ^]^ When bulk Te was reduced to monolayer, a Te atom of in one triangle chain unit moves to the adjacent chain to form mirror symmetry and the structure is termed as *β* phase (shown in **Figure** **28**c).^[^ ^145^ ^]^ With similar crystal structure with BP, the chalcogens also possess many properties analog to BP, such as high anisotropy and tunable bandgap, strong optical absorption, and efficient catalytic activity.^[^ ^146^ ^]^ Moreover, these materials also possess many physical and chemical properties superior to BP, such as better environmental stability,^[^ ^147^ ^]^ high carrier mobility, and can be synthesized by various facile methods.

#### 5.3.2 Applications in Catalysis

Many 2D BP analog monoelemental materials have been theoretically predicted, but few has been reported in related to their photo/electroapplications. Because the synthesis of ultrathin and high‐quality layers still remains a big challenge. Pumera’ group^[^ ^148^ ^]^ has proposed a liquid exfoliation method with kitchen blenders to prepare As, Sb, and Bi nanosheets. The bulk As, Sb, and Bi are stirred and cut in kitchen blenders in the presence of surfactant sodium cholate. The liquid turbulence and shear force result in the exfoliation of bulk pnictogens. Then the sediments were washed and centrifugated to remove the large size particles and the 2D pnictogens nanosheets were obtained. The lower overpotential potentials for HER and OER have been observed in few layer pnictogens compare to the corresponding bulk pnictogens, indicating an improved electrochemical performance of exfoliated pnictogens. Interestingly, the Sb nanosheets exhibit the best electrochemical activity in both HER and OER among the other pnictogens, which shows its promising potential for electrocatalytic applications. The electrolysis H_2_ production usually works in acidic or alkaline condition, in which the HER proceed in fast kinetic. This harsh condition will raise the risk of degradation and limit their long‐term catalytic operation. In this regard, Li and co‐workers^[^ ^149^ ^]^ have developed a neutral‐electrolyte system for HER by using RuS_2_ decorated antimonene as electrocatalyst. The RuS_2_ nanodots were in suit grown on antimonene by sonication at 500 °C for 2 h. Then abundant Ru—S—Sb bonds were formed at the interface between antimonene and RuS_2_. According to the DFT calculation, the free energy potential for HER is −0.21, −0.1, and −0.07 V for RuS_2_, Pt, and Ru—S—Sb/antimonene, respectively. Moreover, the Ru—S—Sb bond would promote the strong orbital interaction between Ru and H, which will give rise to the H adsorption and thereby decrease the HER thermodynamic barrier. As a result, the Ru—S—Sb/antimonene hybrid exhibits a high HER activity in neutral medium. The Ru—S—Sb/antimonene composite exhibits an onset potential is −89 mV in 1.0 m phosphate buffered saline (PBS) and the overpotential is only 153 mV at the current density of 10 mA cm^−2^, which is much outperformed than that of the Pt/C. Further, the Ru—S—Sb/antimonene also presented an excellent durability and stability after 40 h continuous running.

Besides its efficient electrocatalytic activity and excellent stability under ambient conditions, Sb also exhibits a tunable bandgap (1–1.5 eV) which can be modulated for optical absorption and photocatalytic applications. One of the efficient methods to modify the gap is to hybrid Sb with different molecules and materials. Barrio et al.^[^ ^150^ ^]^ have synthesized a 2D–2D heterostructure between graphitic carbon nitride (g‐CN) and antimonene nanosheets (CNSb*_x_*). The CNSb*_x_* heterostructures exhibit a type I heterojunction, where the photoexcited electrons on g‐CN will migrate to the conduction band of Sb while the photogenerated holes will transfer to the valence band, as shown in Figure 28. The degradation of RhB is mainly contributed from the holes on the valence band of CN. The rhodamine B (RhB) dye was fully degraded in only 20 min with the presence of CNSb_0.25_ under visible light irradiation, while the pure Sb shows negligible photodegradation activity. Hybriding the 2D monoelement material to architect a heterojunction can be further adopted to suppress the electron–hole recombination rate. Li et al.^[^ ^151^ ^]^ introduced Se in Sn to design a SnSe_2_/Se heterojunction via a solvothermal route. According to the transient photovoltage (TPV) analysis, the positive photovoltage in SnSe_2_ nanosheets turn into negative in SnSe_2_/Se heterojunction, which indicates the accumulation for electrons. As more electrons are trapped on the surface of SnSe_2_/Se heterojunction, the holes can survive the recombination to induce the oxidation reaction. As a result, the photocatalytic activity is much enhanced with 94% rhodamine Blue (RhB) degradation in 50 min, which is much improved than the pure SnSe_2_ (with 83% degradation) under the same conditions. Additionally, 2D Te is usually used in photocatalytic nitrogen fixation due to its strong nitrogen absorption. Ding et al.^[^ ^152^ ^]^ have hybrid tellurium with graphitic carbon nitride to form a TeCN. With the introduce of 1% Te, TeCN exhibits a nitrogen fixation rate as high as 7.2 times than that of graphitic carbon nitride (CN) under visible light irradiation. This can be ascribed to the Te's better chemical adsorption of N_2_. When receive the photogenerated electrons, Te^6+^ cations could transform to Te^4+^, which would react with N_2_ to form Te^6+^ in turn, which further increased the nitrogen fixation rate. Moreover, as higher CB potential may contributes to a stronger photocatalytic reduction ability,^[^ ^153^ ^]^ the doping of Te raise the CB potential of the catalyst to a more negative level, (0.25 eV negative than that of pristine CN), thus greatly enhanced the nitrogen fixation efficiency by converting nitrogen into ammonia.

## 6 Summary and Perspective

This review summarized recent progress in BP and BP‐analog materials for the photo/electrocatalysis applications. For BP, the highly tunable band structure along with broad‐band absorption, superior and anisotropic carrier mobility as well as abundant intrinsic catalytic active sites totally render BP application prospects in both photo/electrocatalysis fields rather than being limited in only one field. Even though BP owns tremendous opportunities, there are still some challenges for the application of BP in photo/electrocatalysis.

The bottleneck to the BP in catalysis is the poor carrier separation efficiency and degradation nature in catalytic conditions. So far, as summarized above, various efforts such as size control, heterojunction construction, and group functionalization, have been made to improve the photo/electrocatalysis efficiency and ambient stability of BP. Actually, these structure and surface engineering strategies achieving such laudable progress for the enhanced photo/electrocatalytic performance should be mainly attributed to the flexibility of BP to couple with metals, functional groups, acceptor molecules, and other catalysts as well as the highly tunable band structure. The uncoordinated lone pair electrons of P atom and unavoidable point defects especially at edge sites contribute to the chemical activity to interact with heteroatoms, making BP easy to form bond with metals, groups, and even another semiconductor such as g‐C_3_N_4_, MoS_2_. Accordingly, although the intrinsic chemical activity of BP brings about ambient degradation, it inversely induces the engineering flexibility and surface catalytic activity. Thus, it is a perspective approach to overcome the limitations of BP by selecting specific metals or surface groups with catalytic activity to not only passivate BP for enhanced stability, but also become electron acceptors for effectively elongating the lifetime of excited carriers. In addition, designing Z‐scheme heterostructure based on these passivated BP can be an ideal way to further acquire highly stable and efficient photocatalyst.

Although these strategies can be theoretically effective, the preparation technique is still restricted. Owing to the difficulty of synthesis BP through bottom‐up method, most of these BP‐based catalysts are synthesized by ball‐milling method or electrostatic self‐assembly instead of in situ growth and coordinate self‐assembly, both of which are more inductive to form homogeneously interfacial coupling. Thus, exploiting bottom‐up methods, such as solvothermal method, to facilely prepare layer and size controllable BP as well as the aforementioned kind of BP‐based catalysts on a large scale make great sense for the future commercial application.

Ignited by the study of BP, significant progress has been made on the BP‐analog materials, such as 2D binary IV–VI compounds, layered metal phosphite materials and 2D monoelemental materials. These BP‐analog materials share the similar atomic and electro structure with BP as well as outstanding catalytic performance which can be desirable substitutes or complements for BP. 2D binary IV–VI compounds, particularly SnS, exhibits a tunable dual indirect and direct bandgaps with a corresponding strong absorption coefficients across a VIS–NIR region. Surprisingly, SnS exhibits a perfect intrinsic bandgap structure for full water splitting, while BP can only accommodate a half water splitting reaction. All these merits make SnS a promising photocatalyst for future applications. Layered metal phosphite materials, including binary compounds MP*_n_* and thiophosphite materials MPX*_n_*, extensively expanded the family of BP‐analog materials due to the multiple constructions by various M or X elements. Compared to other MPS*_n_* materials, FePS exhibits an excellent stability in a wide PH range from alkaline to acidic conditions, which is a great advantage for long term catalytic applications. However, preparing ultrathin and high‐quality metal phosphite materials still remains a big challenge. As cousins of phosphorene, 2D BP analog monoelemental materials, such as pnictogens and chalcogens, share the same structure with black phosphorus. Similar to BP, the 2D pnictogens (As, Sb, Bi) possess a broad range of bandgap, from 0.36 to 2.62 eV, providing unprecedented opportunities for catalytic applications. Moreover, these materials can be easily obtained from their bulk counterparts or as deposited by various methods like PVD, liquid‐exfoliation, which is of great advantage for industrialization. However, compared to BP, few reports of 2D BP analog materials have been seen on the catalytic applications. Therefore, there is a big room to explore related applications in catalysts for 2D BP analog materials.

From the opinions of the authors, future works can concentrate on the improvement of the stability of BP, functionalizing the BP surface by taking advantages of its intrinsic defects, as well as the construction of BP‐based Z‐scheme heterostructure to achieve the efficient carrier separations. Moreover, the potential of BP‐analog materials on photo/electrocatalytic applications could be further explored under the guidelines from BP studies.

## Conflict of Interest

The authors declare no conflict of interest.
